# Excited-State Relaxation Pathways of 4‑Aminobiphenyl-2-Pyrimidine Derivatives: An Ultrafast Perspective

**DOI:** 10.1021/acs.jpca.5c06504

**Published:** 2025-10-31

**Authors:** Alejandro Cortés-Villena, Soranyel Gonzalez-Carrero, Carolina Aliaga, Moisés Domínguez, Matías Vidal, Pablo Rojas, Raquel E. Galian, Julia Pérez-Prieto

**Affiliations:** † Instituto de Ciencia Molecular, 16781 Universidad de Valencia, Valencia 46980, Spain; ‡ Department of Chemistry and Centre for Processable Electronics, 4615 Imperial College London, London W12 0BZ, U.K.; § Facultad de Química y Biología, 28065 Universidad de Santiago de Chile, Santiago 9170022, Chile

## Abstract

Push–pull systems are key molecular architectures widely studied for their unique photophysical properties and tunable excited-state dynamics. Here, we present a systematic investigation of the influence of (i) solvent environment and (ii) donor-group substituents on the excited-state relaxation pathways and dynamics of two previously reported push–pull systems, namely 4-[4-(4-*N*,*N*-dimethylaminophenyl)­phenyl]-2,6-diphenylpyrimidine and 4-[4-(4-*N*,*N*-diphenylaminophenyl)­phenyl]-2,6-diphenylpyrimidine for **D1** and **D2**, respectively. Previous findings demonstrated the presence of a twisted intramolecular charge transfer (TICT) state, which determined the fluorescent properties of **D1** in highly polar solvents. However, this state was absent in **D2** due to conformational constraints. By integrating steady-state emission mapping, time-correlated single photon counting (TCSPC), and femtosecond/nanosecond transient absorption spectroscopy (fs/ns-TAS), we are able to fully disentangle the complex landscape behind the excited state relaxation pathways in these push–pull systems both in nonpolar and polar solvents. Additionally, the correlation between multiple excited-state components and factors such as viscosity and solvent polarity is thoroughly rationalized. Altogether, these findings shed more light on the complex interplay between molecular conformation and solvent polarity in push–pull systems, which provides valuable insights into the rational design of advanced optoelectronic and photonic materials.

## Graphical abstract

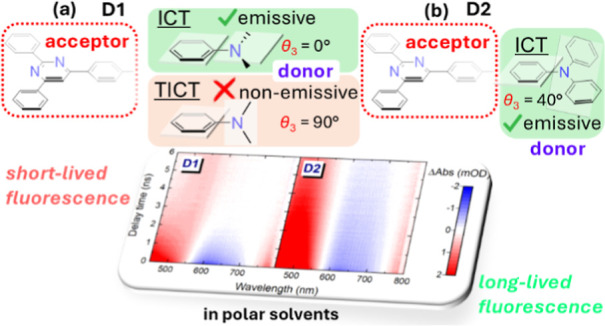

## 1 Introduction

Organic molecular materials that exhibit push–pull (donor–acceptor) electronic characteristics have attracted significant interest for optoelectronic applications due to their tunable photophysical properties, driven by an intramolecular charge transfer (ICT) process.^1^ Upon photoexcitation, the lowest excited state in donor–acceptor systems can manifest in one of three forms: a locally excited (LE) state, a charge transfer (CT) state, or a hybrid state that exhibits characteristics of both LE and CT (HLCT).^2^ While the LE state remains largely unaffected by solvent polarity due its small dipole moment, the CT state is strongly solvent-dependent due to the strong interaction between solvent and molecule.^3,4^ Specifically, the ICT process induces polarization in push–pull systems, resulting in a significantly larger dipole moment in the excited state (ES) relative to the ground state (GS), which can easily be modulated. Therefore, by strategically modifying electron donor (push) and electron acceptor (pull) groups, as well as the π-conjugated linkers, the ICT response can be finely tuned, enabling the design of tailored materials for diverse applications ranging from photovoltaics and light-emitting devices,^5–7^ to (bio)­sensing and nonlinear optical applications,^8,9^ among others.

The optical and photophysical properties of push–pull systems, such as absorption and emission maxima, fluorescence quantum yields, and excited-state lifetimes, are thus strongly dependent on the nature and extent of the ICT in the excited state.^10^ Both the molecular structure of donor/spacer/acceptor groups and surrounding environment (solvent) are critical in determining their functional behavior.^11–14^ Previous studies on structure–property relationship have shown that the nature of the electron donor group, particularly those involving *N*,*N*-dimethylamino (DMA) and *N*,*N*-diphenylamino (DPA) groups, plays a particular role as these groups exhibit distinct electronic and steric effects that modulate charge transfer efficiency, emission behavior, and also conformational dynamics.^13,15–17^ Of particular interest is the sensitivity of fluorescence properties to different external stimuli, as the ICT-based fluorescence response inherently carries implicit information about the surrounding environment,^18^ making them especially valuable for the development of responsive materials for sensing and imaging applications.^19^

Ultrafast time-resolved spectroscopic studies have demonstrated that solvent reorganization around dipolar ICT states contributes to dynamic Stokes shifts, reflecting the evolving stabilization of the excited state by the solvent reorganization dynamics (SD).^20,21^ In many cases, these solvent relaxation time constants have been found to correlate well with ultrafast ICT processes.^22,23^ Building on this understanding, a previous work examined the excited-state ICT behavior of four tetrahydro[5]-helicene-based imide derivatives (THHBI) with different electron-donating groups across solvents of varying polarity, where the strongest donor, THHBI-PhNPh_2_, underwent faster ICT relaxation in more polar solvents, leading to the formation of a more solvent-stabilized ICT′ state.^11^ Excited-state relaxation dynamics of triphenylamine-modified vinylbenzothiazole derivative (BTTM) involved the population of a hybridized local and charge-transfer (HLCT) state.^2^ While the excited state predominantly exhibits LE character in nonpolar solvents, the state evolves into a charge-transfer (CT)-dominated HLCT state as solvent polarity further increases. An additional intermediate HLCT′ state was found in highly polar solvents, indicating a more complex relaxation pathway governed by solvent–solute interactions. Introducing a hydrogen bond was found to restrict rotational motion in the excited state and thus boosted fluorescence quantum efficiency, as demonstrated by the excited-state relaxation behavior of biphenyl derivatives (TPA-PPI and TPA-PPI–OH) in different solvents.^24^

Pyrimidine-based chromophores have emerged as versatile building blocks in photophysics and optoelectronics owing to their electron-deficient character, modular synthesis, and compatibility with donor–acceptor (push–pull) design. These scaffolds readily support efficient ICT and have been implemented across emissive and sensing platforms, including OLEDs and responsive probes.^6,8,25,26^ In parallel, the role of the aryl linker is decisive: biphenyl/biphenylene bridges extend π-conjugation while introducing a tunable dihedral angle that governs electronic coupling and the populations of excited-state conformers, thereby modulating radiative vs nonradiative decay channels and, ultimately, fluorescence efficiency.^25,27–30^ Recent studies explicitly correlate donor–acceptor torsion with the balance between HLCT and TICT states, oscillator strength, and singlet–triplet energy gaps, underscoring that controlling linker planarity/rigidity is a powerful strategy to enhance emission in push–pull systems.^27,28,30^ These broader insights motivate our focus on pyrimidine acceptors with biphenyl linkers to disentangle how solvent polarity and conformational degrees of freedom dictate the excited-state relaxation landscape.

After photoexcitation, rotation around critical dihedral angles, can generate in principle distinct conformers and thus significantly affecting the emission yield and its energy.^22,31–33^ In the excited state, rotation around a single bond can reduce the system’s energy by forming a nonemissive twisted intramolecular charge transfer (TICT) state, enabling an additional nonradiative excitation decay channel, which may not be present if this twisting is forbidden by, for instance, steric hindrance.^19,34–37^ This reduced fluorescence quantum yield of push–pull systems in highly polar solvents owing to electronic wave function decoupling has widely been attributed to the population of this dark state.^38–41^ Despite tremendous efforts over the past decades, including the design of numerous model compounds and the use of diverse spectroscopic methods, clear experimental confirmation still remains elusive. Moreover, the significance of TICT states continues to be a hot topic of debate, reflecting ongoing uncertainty about their role in photophysical and photochemical processes and underscoring the need for further theoretical and experimental investigation.^42^

In a previous study,^43^ we rationalized the emissive properties of two push–pull systems, namely 4-[4-(4-*N*,*N*-dimethylaminophenyl)­phenyl]-2,6-diphenylpyrimidine and 4-[4-(4-*N*,*N*-diphenylaminophenyl)­phenyl]-2,6-diphenylpyrimidine for **D1** and **D2**, respectively (Scheme 1). We found that the torsion angle of the disubstituted amino group, whether *N*,*N*-dimethylamino or *N*,*N*-diphenylamino, at the biphenyl terminus was decisive on the emissive properties of the molecule. The identification of a TICT state in **D1**, which was absent in **D2**, as revealed by both experimental and computational analyses, provided a comprehensive explanation for the emission efficiency.^43^ However, early time events following photoexcitation on such push–pull systems in different solvents remained still unexplored, which motivated us to go more in depth in that direction and hence is the main focus of the present work. Here, we conducted transient absorption measurements (from femto to nanosecond time scale) aimed at understanding the excited-state deactivation pathways and dynamics of **D1** and **D2** push–pull systems in four selected solvents, that is, hexane, toluene and acetonitrile, dimethyl sulfoxide as nonpolar and highly polar solvents, respectively. Ultrafast spectroscopic analyses uncovered the presence of multiple excited-state species arising from distinct molecular conformers, each exhibiting lifetimes that are strongly modulated by the viscosity and polarity of the solvent. While the emissive state of **D1** becomes progressively shorter-lived with increasing solvent polarity, consistent with the formation of a TICT state, an assignment also supported by kinetic target modeling, the absence of this TICT state in **D2** owing to conformational rigidity, resulted in a significantly longer-lived emission even in highly polar solvents. Collectively, these findings demonstrate the critical influence of both the solvent and molecular conformation on the population and dynamics of excited-state relaxation pathways.

**Scheme 1:**
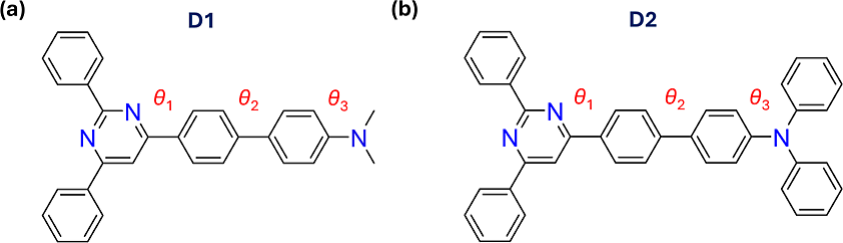
Molecular Structures of (a) **D1** and (b) **D2** Push–Pull Systems^a^

a Key dihedral angles relevant to molecular conformation are highlighted.

For the sake of a clearer comparison, we focus on **D1** in hexane versus **D1** in acetonitrile throughout the manuscript since they exhibit markedly different solvent polarities, yet are similar in terms of viscosity, while data for **D1** in other solvents and for **D2** in all solvents are provided in the Supporting Information.

## 2 Experimental Section

### 2.1 Chemicals

**D1** and **D2** push–pull systems were synthesized according to previously reported methods.^44^ All solvents (*n*-hexane: HX and toluene: TOL as nonpolar solvents; acetonitrile: ACN and dimethyl sulfoxide: DMSO as highly polar solvents) were purchased from Merck (>95% for HX and >99% for rest) and used as received without further purification.

### 2.2 Steady-State Absorption and Emission Spectroscopy

Absorption spectra were recorded on a UV/vis/NIR PerkinElmer Lambda 1050 spectrophotometer equipped with deuterium and tungsten halogen light sources and a photomultiplier tube (PMT) detector covering from 300 to 800 nm. Emission spectra were recorded on a FLS1000 photoluminescence spectrometer (Edinburgh Instruments) equipped with a 450 W ozone free continuous xenon arc lamp and a PMT-980 detector in cooled housing with extended spectral range from 380 to 800 nm. All measurements were conducted under anaerobic conditions. Phosphorescence measurements were carried out under liquid nitrogen conditions. For singlet oxygen measurements, a NIR-PMT detector (Hamamatsu Photonics) with a spectral response ranging from 1000 to 1500 nm was employed. The samples were bubbled with pure oxygen for 15 min in quartz cuvettes with optical path length of 1 cm. All spectra were recorded using a right-angle geometry and corrected for the instrument’s spectral sensitivity and fluctuations in lamp intensity.

### 2.3 Spectroelectrochemistry

Spectroelectrochemical (SEC) measurements were carried out using a home-built system equipped with an OceanOptics HL-2000-FHSA halogen light source and a Maya2000Pro spectrometer while applying controlled potentials with a Metrohm Autolab PGSTAT101 potentiostat. SEC absorption difference spectra were acquired by increasing the applied potential in steps of 100 mV vs the Ag/AgCl reference electrode and acquiring a transmittance spectrum (500–900 nm) at each applied potential. Absorbance difference spectra were calculated versus a transmittance spectrum taken at 0 V vs Ag/AgCl. Data acquisition was managed by using LabView software, with five consecutive spectra averaged at each potential.

### 2.4 Time-Correlated Single Photon Counting

The same samples for steady-state measurements were used for time-resolved measurements using the time-correlated single photon counting (TCSPC) technique under deoxygenated conditions. A 375 nm picosecond pulsed-laser (EPL-375) with a repetition rate of 10 MHz as excitation source and a microchannel plate (MCP-900) detector in a cooled housing with a spectral range of 380–800 nm as detector were used. For instrument response function (IRF), a Ludox solution in water (0.1 OD at the excitation wavelength) was employed, and was approximately 120 ± 10 ps in our setup. TCSPC histograms were collected up to 3000–5000 counts as stop conditions (peak count) and were fitted with Fluoracle software to multiexponential decay functions by reconvolution methods.

### 2.5 Femtosecond Transient Absorption Spectroscopy

Femtosecond transient absorption spectroscopy (fs-TAS) measurements in the femtosecond-to-nanosecond time domain were conducted using an Agilent Helios spectrometer (Spectra Physics, Newport Corp.). Ultrafast laser pulses (800 nm, 100 fs, 1 kHz) were generated by a Ti:sapphire regenerative amplifier (Solstice, Spectra Physics). The output beam was split into pump and probe paths. The pump beam was tuned to 355 nm with 100 nJ/pulse using an optical parametric amplifier (TOPAS Prime) coupled with a frequency mixer (Niruvis, Light Conversion) and modulated at 500 Hz by a mechanical chopper. The probe beam was directed through a mechanical delay stage (time window: 6 ns) and focused into a sapphire crystal to generate a white-light continuum spanning 450–800 nm. The probe was split into reference and signal paths using a neutral density filter and directed to the sample and reference detectors, respectively. Both signals were collected by fiber-optic coupled multichannel spectrometers equipped with Si and InGaAs sensors. Pump and probe beams were spatially and temporally overlapped at the sample position. All samples were bubbled with argon for 10 min in quartz cuvettes with optical path length of 0.2 cm, using solutions with absorbances of 0.5 OD at the pump wavelength. Data analysis was carried out using Surface Xplorer software (version 4.3.0, Ultrafast Systems). The data was first preprocessed by cropping the range, subtracting background, by chirp correction, and globally analyzed using GloTarAn software.^45^

### 2.6 Nanosecond Transient Absorption Spectroscopy

Nanosecond transient absorption spectroscopy (ns-TAS) in the nanosecond-to-microsecond time domain was performed using deoxygenated samples on a laser flash photolysis (LFP) spectrometer (LP980-KS, Edinburgh Instruments) equipped with a Quanta-Ray INDI Nd:YAG laser (Spectra Physics) with a parametric optical oscillator (primoScan BB, Spectra Physics). The pump wavelength and energy/pulse were fixed to 355 nm (third harmonic of the laser) and 5 mJ/pulse for all samples. Sample solutions were fixed to 0.5 OD at pump wavelength. The IRF was 10 ns with this setup. A 150 W Xe pulsed lamp for probing the excited-state species. For spectral and kinetic measurements, an ICCD camera (Andor DH320T) and a PMT were used, respectively. The L900 software was used to fit the kinetic data by tail fitting methods.

### 2.7 Kinetic Model

All processed fs-TAS data was imported into GloTarAn software,^45^ a graphical interface built on the TIMP package in R,^46,47^ for global and target analysis. Initially, a fully sequential/parallel kinetic model was applied to evaluate the number of components required to describe the system. Once the minimum number of components was determined, a branching kinetic model (reflecting the proposed excited-state relaxation pathways) was constructed for target analysis. Global fitting was performed using a nonlinear least-squares algorithm with 10 iterations, and convergence was ensured by evaluating both the left and right singular vectors of the residual matrix and the root-mean-square (RMS) of the residuals. Then, the result of the target analysis yielded species-associated difference spectra (SADS), which represents the true spectral signatures of individual kinetic species and their associated lifetimes. This approach enabled the resolution of overlapping spectral features such as the TICT state and the assignment of distinct kinetic components to specific excited-state species.

## 3 Results and Discussion

### 3.1 The Dual Emission

The normalized steady-state absorption and fluorescence spectra of diluted solutions (0.1 OD at 365 nm) of **D1** and **D2** push–pull systems were investigated in four solvents spanning a wide polarity range (*n*-hexane: HX and toluene: TOL as nonpolar solvents; acetonitrile: ACN and dimethyl sulfoxide: DMSO as highly polar solvents) at room temperature and under anaerobic conditions (Figure S1). Absorption maxima were slightly affected (349–376 nm) while fluorescence maxima are strongly red-shifted (403–609 nm) by increasing the solvent dielectric constant (ε) from 1.9 to 46.7, indicating the low and high dipole moment in the ground and excited state, respectively. This corroborates the nature of the intramolecular charge transfer (ICT) of the emitting state rather than formation of an aggregate state.^48^

The slight differences in absorption spectra are mostly related to different geometries in the ground state.^49^ Notably, the vibronic fine-structure of both **D1** and **D2** systems in HX is noteworthy, arising from the negligible molecule–solvent interactions. The fluorescence quantum yields (Φ_F_) for **D1** and **D2** in HX are 87 and 75%, respectively. The emergence of two emission bands (dual emission), likely determined by the different nature and geometry of the transition, is discernible in highly polar solvents. In TOL, the Φ_F_ of **D1** and **D2** remain high at 87 and 96%, respectively. Notably, the dual emission of **D1** is clearly apparent in ACN (19%) and DMSO (16%), whereas this feature is less pronounced for **D2** (78 and 79%, respectively). This behavior can be attributed to the relatively low fluorescence quantum yield (Φ_F_) of the ICT band of **D1** in polar solvents compared to that of the highest-energy one, as demonstrated in our previous study.^43^

To further investigate this dual emission, first we conducted emission mapping experiments of **D1** under varying excitation wavelengths (from 260 to 410 nm, each 10 nm) to undoubtedly visualize the population of the high-energy emitting band at lower excitation wavelengths (Figure 1a,b). The molecule **D1** in HX exhibits an identical fluorescence spectrum across the selected excitation wavelengths (maximum center at 403 nm), characterized by a fingerprint-type single band. In contrast, **D1** in ACN displays two distinct emission bands, being the ICT band (maximum at 593 nm) the most intense band. While **D1** in HX does not appear to populate an intramolecular charge transfer (ICT) state, **D1** in ACN exhibits a broad, red-shifted emission band at 593 nm alongside a high-energy emitting band between 360 and 460 nm, especially when excited below 350 nm. Based on the position of this high-energy emitting band and the low dipole moment of the ground state, we assign it to a locally excited (LE) state.^27,50,51^

**Figure 1:**
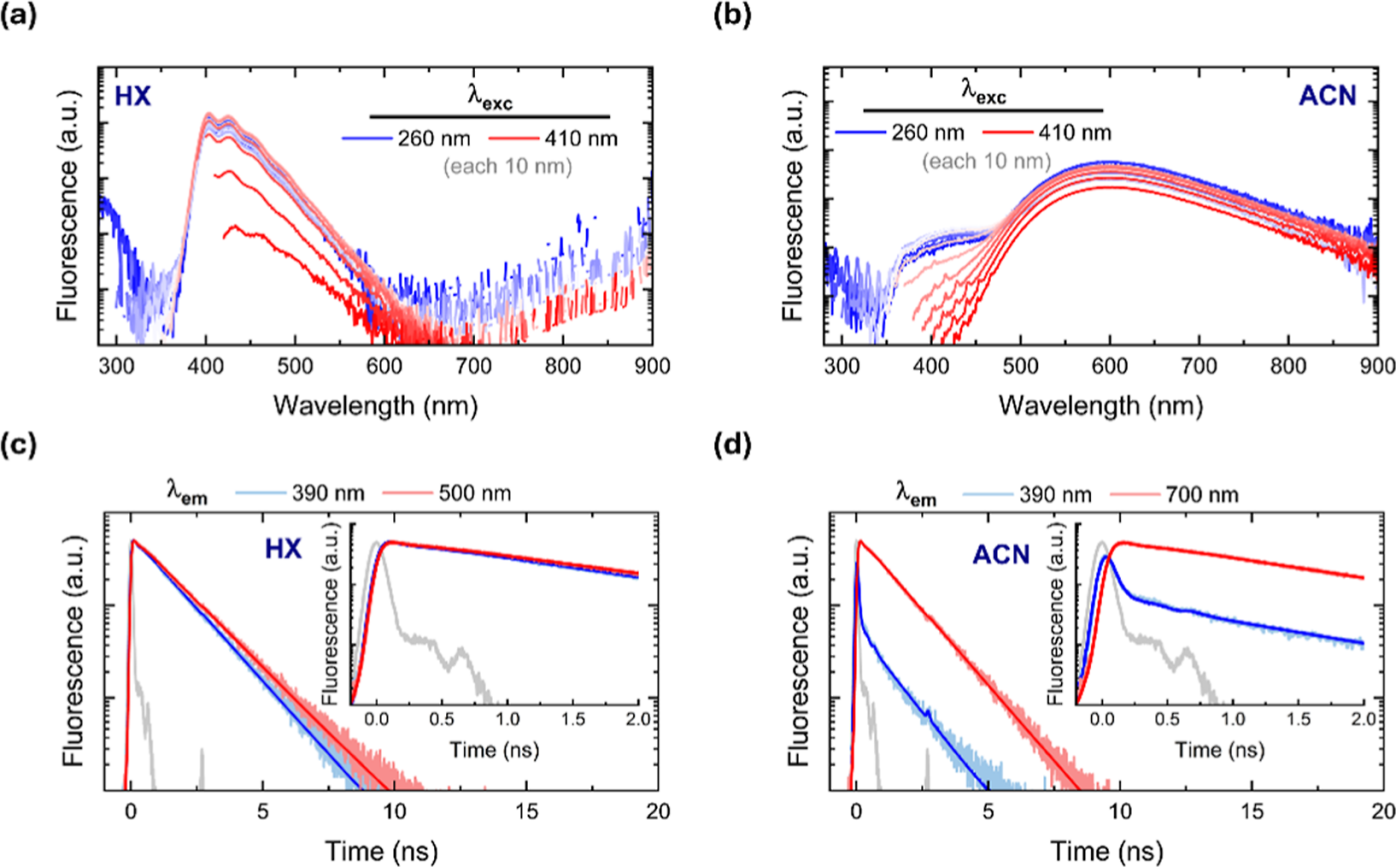
Steady-state fluorescence spectra of (a) **D1** in HX and (b) **D1** in ACN under varying excitation wavelengths (260–410 nm; each 10 nm). Fluorescence in log scale. Fluorescence decay kinetics for (c) **D1** in HX at 390 and 500 nm and for (d) **D1** in ACN at 390 and 700 nm under 375 nm picosecond pulsed-laser excitation (inset: early time trace).

We next turned to explore the fluorescence decay kinetics by time-correlated single photon counting (TCSPC) technique upon picosecond pulsed-laser excitation at 375 nm and recorded at wavelengths at which either LE or ICT transition occurs (Figure 1c,d).^52^ In hexane, **D1** shows negligible differences in the emission decay traces at the two emission wavelengths explored (lifetime 1.4–1.5 ns), indicative of the same emitting state. By contrast, **D1** in ACN exhibited clear differences in the emission decay. First the 390 nm band shows a fast decay (limited by the temporal resolution of our TCSPC setup, ca. 120 ps) accompanied by a slow decay of 1.2 ns. The 700 nm band, on the other hand, exhibits a rise at 700 nm (see Figure 1d, inset for the time lag), followed by a decay with a lifetime of 1.3 ns.^53,54^ Even shorter lifetime of the ICT state (for instance, 0.9 ns at 700 nm in DMSO, Figure S3) supports the preferred population of a nonemissive twisted intramolecular charge transfer (TICT) state for **D1** in more polar solvents and, in turn, facilitate nonradiative transitions– a feature absent in **D2**– as evidenced by the long-lived fluorescence of its ICT band of 4.6 and 4.3 ns at 700 nm in ACN and DMSO, respectively (Figures S4 and S5).^55^ The long-lived fluorescence in highly polar solvents indicates stabilization of the ICT state. Good fitting of the kinetic traces was obtained and all parameters are summarized in Table S1.

To further examine the role of molecular geometry on the dual-emission behavior, we performed additional fluorescence measurements of **D1** in polar solvents of different viscosities (Figure S2). In viscous media such as glycerol, torsional relaxation is hindered, resulting in emission predominantly from a geometry resembling that of the ground state and exhibiting a longer-lived emission. A minor contribution from a lower-energy emission (emission tail beyond 600 nm) is noted. Time-resolved fluorescence measurements at 488 nm revealed two decay components of 1.3 ns (70%) and 4.3 ns (30%), indicating that the dominant contribution arises from the higher-energy emitting band. Conversely, in less viscous solvents like MeOH, the twisting in the excited state gives rise to a clear additional lower-energy band around 600 nm, consistent with a more planar and stabilized structure, as supported by our previous calculations.^43^ The kinetic trace at 488 nm shows substantial quenching: the first component is time-limited by the IRF, while the second one has a lifetime of 1.3 ns (64%). These observations confirm that the more planar and stabilized structure is directly connected to the TICT state, and only emission from the higher-energy torsionally relaxed structure is observed.

Time-resolved emission spectra (TRES) mapping (recorded every 0.4 ns from 380 to 800 nm in TCSPC mode) reveal distinct excited-state behaviors for **D1**, particularly in relation to solvent polarity (Figure S6). In nonpolar solvents, where the TICT state is not accessible, **D1** exhibits minimal spectral evolution. For example, in TOL only a gradual decrease in the high-energy emission band consistently with the behavior previously shown in Figure S3, and a minimal redshift of the ICT band over time is observed, indicating still weak solvent-dependence of fluorescence. However, in highly polar solvents where the TICT state becomes accessible, more complex behavior emerges. In addition to the initial decay of the high-energy emission band, a concomitant blueshift of the ICT band over time is observed, which differs from the behavior in nonpolar media, suggesting a dynamic population of distinct emissive states (Figure S6). The apparent blueshift of **D1** in ACN and DMSO can be attributed to the earlier decay of the lower-energy emission band than the higher-energy band, resulting from the preferential accessibility of the former to the TICT channel. As a point of comparison, **D2**—lacking a TICT state—shows time-resolved spectral behavior similar to that of **D1** in TOL (Figure S7), where only a decrease of the high-energy band and a slight redshift of the ICT band over time is apparent, reinforcing the role of TICT dynamics in the solvent-dependent response. Gathering all these results together, these findings highlight the complex interplay between multiple excited-state pathways, especially the influence of solvent polarity on the population and relaxation of LE, ICT, and TICT states.

### 3.2 Dependence of Ultrafast Properties on Solvent Polarity and Viscosity

With the aim of assessing early time events and subsequent photophysical transitions of both **D1** and **D2** push–pull systems, we conducted femtosecond broadband transient absorption spectroscopy (fs-TAS) experiments following 355 nm laser excitation (100 nJ/pulse) in the selected solvents. Figure 2a shows the TA spectra of **D1** in HX at indicated delay times (0.3–6000 ps) probed in the visible range (450–800 nm). At early delay times (0.3 ps), the TA spectrum exhibits a pronounced excited-state absorption (ESA), featured by two distinct peaks at 495 and 566 nm. Over time, the TA spectral shape remains largely unchanged, reaching the maximum at 3 ps, with only a slight decrease in intensity observed in the short-wavelength region (<460 nm).^56^ This minimal spectral evolution is consistent with the emission mapping results indicating the LE nature of the excited state as indicative of negligible molecule–solvent interaction.^57^ Figure 2b displays the kinetic traces extracted at two representative wavelengths, that is, 456 and 500 nm, including multiexponential function fitting by global analysis (discussed below). The kinetics at 456 nm shows a fast decay in the first 5 ps, concomitantly with a rise in the kinetics at 500 nm, suggesting a precursor–successor relationship from one excited-state species into another. Although the majority of the TA signals constantly decay within the nanosecond time scale, a residual signal (kinetic trace at 500 nm) persists well beyond the temporal resolution of our fs-TAS setup (>6 ns). This long-lived component is likely due to the population of excited-state species with extended lifetimes such as molecular triplets (vide infra) under deoxygenated conditions.

**Figure 2:**
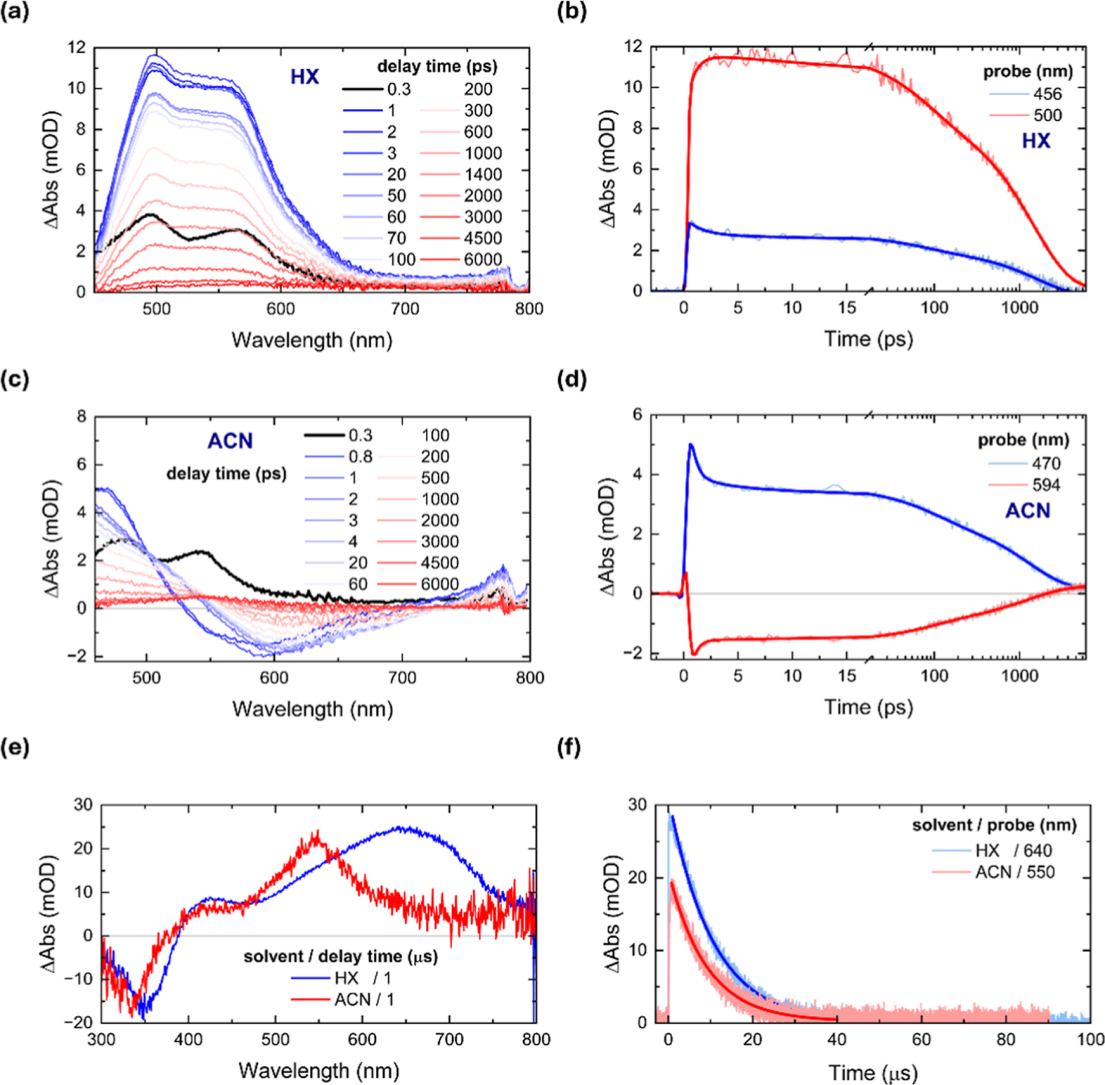
(a) fs-TA spectra of **D1** in HX (λ_pump_ = 355 nm) at indicated delay times and (b) two kinetic traces at two representative wavelengths. (c) fs-TA spectra of **D1** in ACN (λ_pump_ = 355 nm) at indicated delay times and (d) two kinetic traces at two representative wavelengths. (e) ns-TA spectra of **D1** in HX and ACN (λ_pump_ = 355 nm) at 1 μs delay time and (f) kinetic traces at their corresponding maxima. Exponential fittings are included (solid lines) in all kinetic traces.

On the other hand, the TA spectra of **D1** in ACN exhibits pronounced temporal evolution (Figure 2c). First, the ESA at 0.3 ps resembles that in HX, but slightly blue-shifted, with peaks at 481 and 542 nm. This characteristic ESA feature rapidly evolves within 0.8 ps into a new ESA and stimulated emission (SE) features peaking at 468 and 587 nm, respectively. Over time, the SE band undergoes a redshift and intensity decrease, while the ESA band continues to blue-shift– eventually moving outside the visible probe range (<450 nm) and a new absorption feature emerges in the 510–550 nm region. At longer delay times (4.5–6 ns), only a weak signal centered at 545 nm remains, likely arising from long-lived triplet-state populations (vide infra). This dynamic behavior of **D1** in ACN is further illustrated by the kinetic traces shown in Figure 2d, which reveal distinct temporal characteristics and a strong dependence on solvent polarity relative to those found in HX. Notably, the SE band at 594 nm is rapidly formed and decays within the first 3 ps (Figure 2d). Beyond this point, both kinetic traces exhibit a more uniform decay trend. Based on our previous study,^43^ we expect that the formation of the ICT band would be given by the first subpicosecond process (<1 ps).^58^ The changes of **D1** were comparatively minor in TOL due to the lower solvent polarity, but still rapid change of the ESA feature at early times and further ESA feature relaxation (Figure S8). A similar TA spectral evolution as in ACN was observed for **D1** in DMSO although slower due to the increased solvent viscosity. Similarly, the **D2** molecule showed comparable behavior in all studied solvents (see spectra in Figures S9 and S10); however, the associated dynamics differ significantly, highlighting the influence of both solvent polarity and molecular conformation on the excited-state relaxation pathways and dynamics.

To investigate the longer-lived excited-state dynamics of **D1** and **D2** (beyond time resolution of fs-TA setup), we performed nanosecond transient absorption spectroscopy (ns-TAS) in the nanosecond to microsecond time domain. The TA spectra were recorded over the 300–760 nm probe range under both deoxygenated and oxygenated conditions. The ns-TA spectra of **D1** and **D2** at various delay times, along with their kinetic traces at the absorption maxima fitted with single-exponential decay models, are shown in Figures S11–S14 and their fitting parameters are listed in Table S2. For **D1** in HX, the ns-TA spectrum at 1 μs (Figure 2d; Figure S11 for different delay times) displays several distinct features: a ground-state bleach (GSB) at 350 nm and two ESA bands at 420 and 640 nm. The broad ESA centered at 640 nm exhibited a lifetime of approximately 10 μs under deoxygenated conditions, which is significantly quenched to just a few nanoseconds under oxygen-saturated conditions (Figure S15a), assigned to triplet excited state absorption (T_1_ → T_n_). Similar absorption features were observed for **D2** in nonpolar solvents, where triplet-state lifetimes were found to be a way longer than those of **D1**.

The involvement of triplet states typically requires an energetically and orbitally favorable transition to overcome the spin-forbidden nature of the intersystem crossing (ISC) process.^59^ In the case of **D1**, the adoption of either its planar or twisted conformations, as confirmed in our previous study,^43^ may facilitate such transitions by enhancing spin–orbit coupling and thus accessing relaxed triplet states.

Notable is the fact that the ns-TA spectra of **D1** in TOL exhibited a different temporal evolution compared to that in HX (Figure S11c). A redshift of GSB feature upon increasing delay time, together with a band emerging at 700 nm at the expense of the triplet-state decay, was observed. Additionally, the behavior of the 420 nm band was markedly different compared to that in HX, and was found to be largely independent of oxygen species, further supporting that it does not originate from a typical triplet excited state.^60^ In fact, the nature of the 420 nm feature for both **D1** and **D2** in all solvents (except in HX), deviates significantly from the expected behavior of triplet states under oxygen-saturated conditions (Figure S15b).

In order to disentangle the nature of the latter species, we performed spectroelectrochemical (SEC) experiments of **D2** in ACN under applied positive and negative bias using Ag/AgCl as reference electrode. At first glance, the SEC allows one to measure absorption features of radical cations/anions of organic molecules via electrical bias. Under positive bias (1.2. V), that is, slightly higher than the oxidation potential of **D2** previously measured in ACN,^43^ **D2** showed an absorption spectrum with maxima located at 420 and 800 nm as shown in Figure S16a. These absorption features are in good agreement with those of the ns-TA bands described above (ca. 430 and 750 nm); see comparison of ns-TA (at 0.25 μs delay time) and SEC spectra in Figure S16a. The SEC band generated under positive bias (800 nm) confirms the formation of radical cations in these solvents.^61^

Conversely, in highly polar solvents such as ACN and DMSO (Figure S12), the absence of the intense 750 nm band of **D2** in the ns-TA spectrum at 20 μs delay time excludes the presence of radical cations at longer delay times. Instead, the existence of residual ESA around 420–430 nm, along with the redshift of the GSB at longer delay times, suggests the formation of new species, namely radical anions, as observed in the SEC spectra under negative bias (Figure S16b). Interestingly, this behavior occurs only in solvents that can support ICT phenomena (all investigated solvents except HX).

Curiously, no triplet excited states were detected in highly polar solvents (Figure S14), where only a weak absorption band near the radical anion region (430–445 nm) was noted. Therefore, the relaxation of the S_1_ state in **D2** is directly through the radiative and nonradiative transition to the ground state, where the nonradiative transition is the primary pathway of decreasing the fluorescence quantum yield in this molecule. To better contextualize these findings, **D1** in HX and ACN are plotted together at 1 μs delay time (Figure 2e). **D1** in ACN exhibits a significant blueshift of the triplet-related feature, indicating the stabilization of the T_1_ (ICT) state and, consequently, a higher energy separation (T_1_ → T_n_) relative to the T_1_ (LE) state is obtained.^62^ Although **D1** in both HX and ACN solvents yields triplets with comparable lifetimes, the relative amount of triplet populations differs from one another (Figure 2f).

In order to determine the phosphorescence spectra, and thus, the location of the T_1_ energy level for **D1** and **D2** push–pull systems, phosphorescence experiments were carried out at low temperatures (−196 °C). The phosphorescence spectrum of both compounds exhibited a vibronic fine-structure with lowest-energy maxima located at 572 and 552 nm for **D1** and **D2**, respectively, which were found to be ca. 2.2 eV for both compounds (Figure S17). This suggests that triplet energy is negligibly affected by structural changes in the substituents of the donor group. Additionally, singlet oxygen (^1^O_2_) phosphorescence measurements around 1270 nm in the near-infrared (NIR) region serve as an alternative approach to confirm the population of molecular triplets in these systems, given the high thermodynamic driving force to satisfy the following reaction: T_1_ + ^3^O_2_ → S_0_ + ^1^O_2_. Herein, the T_1_ energy (located at 2.2 eV) is much higher than that of the ^1^O_2_ (0.98 eV), thus proceeding this reaction under diffusion-controlled conditions.^63^ For that, these experiments were performed under oxygen-saturated conditions to facilitate efficient Dexter-type triplet energy transfer (TET) from the push–pull molecules to triplet oxygen (^3^O_2_), ultimately leading to the generation of ^1^O_2_. In principle, ^1^O_2_ generation was concluded only for **D1** and **D2** in nonpolar solvents, considering the higher ability to produce triplets (as seen in Figures S11–S14), whereas the ^1^O_2_ emission overlaps with the broad fluorescence tail of the ICT band in highly polar solvents, thereby complicating its detection (Figures S18 and S19).

### 3.3 Target Analysis and Kinetic Modeling

Global target analyses of the transient data matrix for both **D1** and **D2** compounds in the investigated solvents were conducted using a specific kinetic model (shown below), which enabled deconvolution of overlapping spectral features associated with multiple coexisting species and extraction of their corresponding excited-state lifetimes following 355 nm pump photoexcitation. We first applied global analysis in order to identify the minimum number of kinetically distinct components required to adequately describe the temporal evolution of the system. Subsequently, we applied target analysis using specific kinetic models as a second step based on plausible physical and chemical assumptions to yield true component-associated spectra and time constants reflective of the underlying photophysical processes. This built-up the corresponding species-associated decay spectra (SADS) and corresponding population profiles, which are shown in Figure 3 for **D1** in HX and ACN; rest in Figures S20–S25. Global fitting of **D1** in HX revealed four distinct states with lifetimes 2.5 ps, 64 ps, 1.4 ns and 10 μs, which were ascribed to vibrationally hot S_1_ LE [S_1_ (LE*)], S_1_ (LE-1), S_1_ (LE-2), and T_1_ (LE), respectively (Figure 3a,b). The 64 ps and 1.4 ns components were attributed to different conformers of the LE state, suggesting a branch-like mechanism alike to what was pointed out in the quinoid/antiquinoid benzene ring distortions,^64^ with the 1.4 ns lifetime corresponding to the structurally relaxed form, consistent with the fluorescence decay obtained from TCSPC measurements.^32,65^ Conversely, the shortest component (2.5 ps) is tentatively assigned to vibrational cooling within S_1_ manifold, given the clear vibronic structure and absence of charge transfer signatures/solvent reorganization in this completely nonpolar medium.^49^ In contrast, the 10 μs component was fixed based on our previous ns-TA results, due to the limited time window of the fs-TA experiment. In particular, the minimal spectral evolution observed with increasing pump–probe delay time is consistent with the negligible molecule–solvent interactions characteristic of HX, thus confirming the LE nature of the excited state. Even in this nonpolar solvent, four kinetic components are already required to describe the excited-state dynamics. Therefore, in polar solvents, where stronger molecule–solvent interactions and greater stabilization of the excited state occur, it is reasonable that more components are needed to accurately capture the full dynamical behavior.

**Figure 3:**
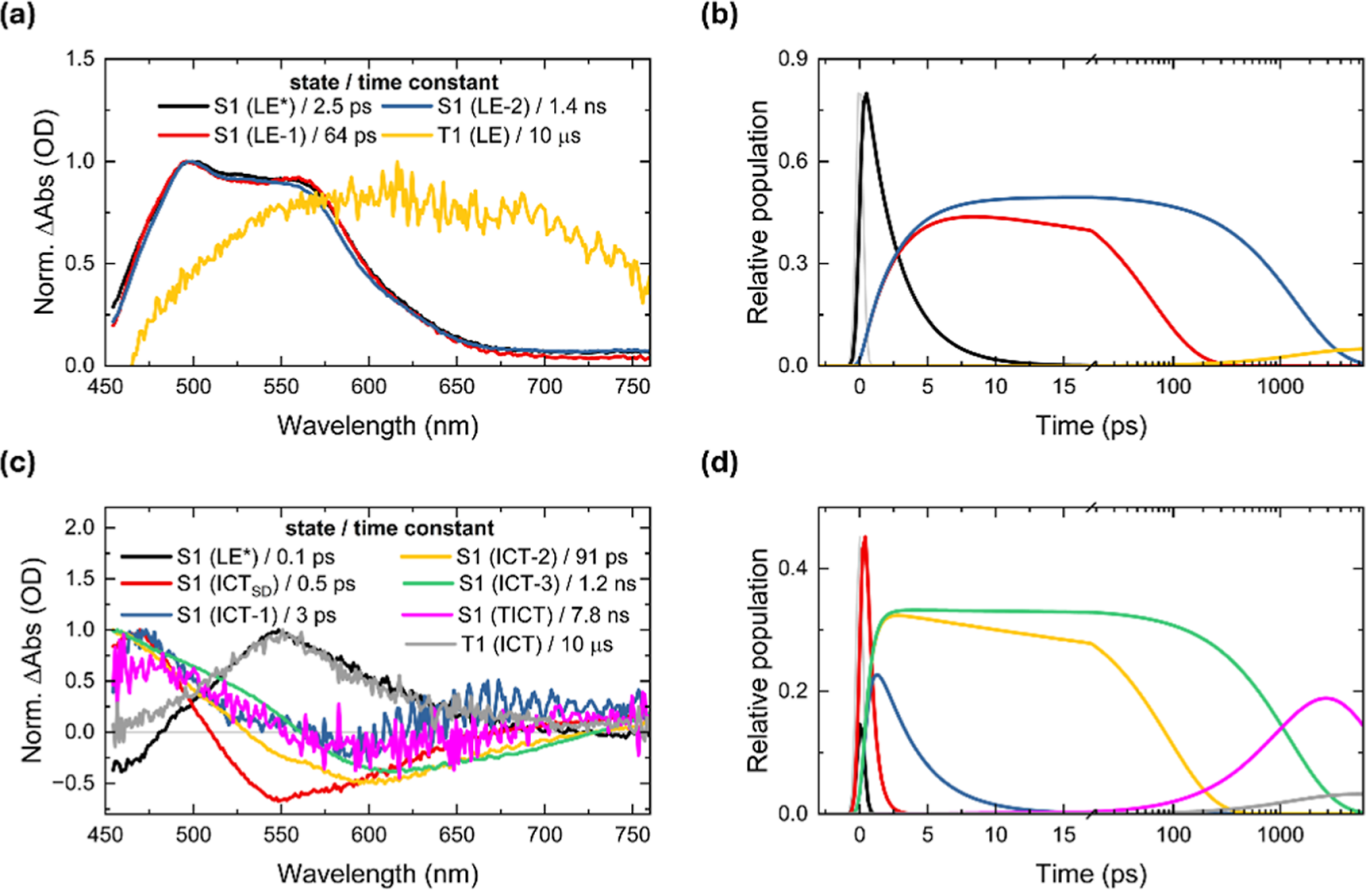
(a) SADS of **D1** in HX along with the time constants obtained through global target analysis and (b) their corresponding population profile over time. (c) SADS of **D1** in ACN along with the time constants obtained through global target analysis and (d) their corresponding population profile over time. SADS are normalized for a better comparison. The IRF for the fs-TAS setup is 0.25 ps.

By contrast, a different scenario is unambiguously presented for **D1** in ACN (Figure 3c,d). Target analysis yielded up to seven states with lifetimes <0.1 ps, 0.5 ps, 1.2 ns, 91 ps, 3 ps, 7.8 ns, and 10 μs, which were in good agreement with the TA spectrum and were reasonably assigned to S_1_ (LE*), S_1_ (ICT_SD_), S_1_ (ICT-1), S_1_ (ICT-2), S_1_ (ICT-3), S_1_ (TICT), and T_1_ (ICT), respectively.^66^ At first glance, the first component (<0.1 ps) was pulse-limited and exhibited a sharp spectral profile, likely reflecting the initially populated LE state, similar to that observed for **D1** in HX, also confirmed by fs-TA spectrum (Figure 2c). The deactivation of this species would correspond to the ICT process from the dimethylamino to the pyrimidine subunit. As delay time increases, a broad SE feature emerges, peaking around 550 nm, which evolves over the next 0.5 ps into progressively red-shifted SE bands, meaning that this ICT state is further stabilized. Therefore, we associate the S_1_ (ICT_SD_) with solvent reorganization dynamics occurring on this characteristic time scale.^67^

Subsequently, three distinct emitting ICT states were resolved, likely associated with different conformers with lifetimes of 3 ps, 91 ps and 1.2 ns with nonidentical emission maxima, as previously discussed.^68^ Owing to the close resemblance of their SADS and the presence of stimulated emission in ACN and DMSO, at least three intermediate components observed for **D1** and **D2** were attributed to different excited-state conformers. At the same time, the latter one (1.2 ns) perfectly matches the longer fluorescence lifetime of the ICT band (Table S1). Implicit is the 7.8 ns component wherein the SE in the 500–750 nm spectral range is absent (within noise) and was reasonably assigned to a TICT state.^69^ Comparable TICT nanosecond lifetimes have been reported elsewhere.^51,70^ Most importantly, this transient spectrum closely resembles a charge-separated state (CSS), as evidenced in Figure S16b. In this configuration, the electronic communication between the donor (dimethylamino group) and the acceptor (pyrimidine group) is significantly disrupted, leading to a decoupling of their electronic states. Therefore, this decoupling is indicative of strong intramolecular charge transfer, where the electron density is effectively shifted from the donor to the acceptor moiety, resulting in the formation of a long-lived, spatially separated excited state.^71^ The last component (10 μs), associated with the T_1_ (ICT) state, was fixed as discussed above. In short, the excited-state dynamics of **D1** in polar solvents like ACN are markedly more complex than in nonpolar environments such as HX, with clear evidence of multiple ICT-related species. The kinetic model used to globally fit the data is shown in Figure 4.

**Figure 4:**
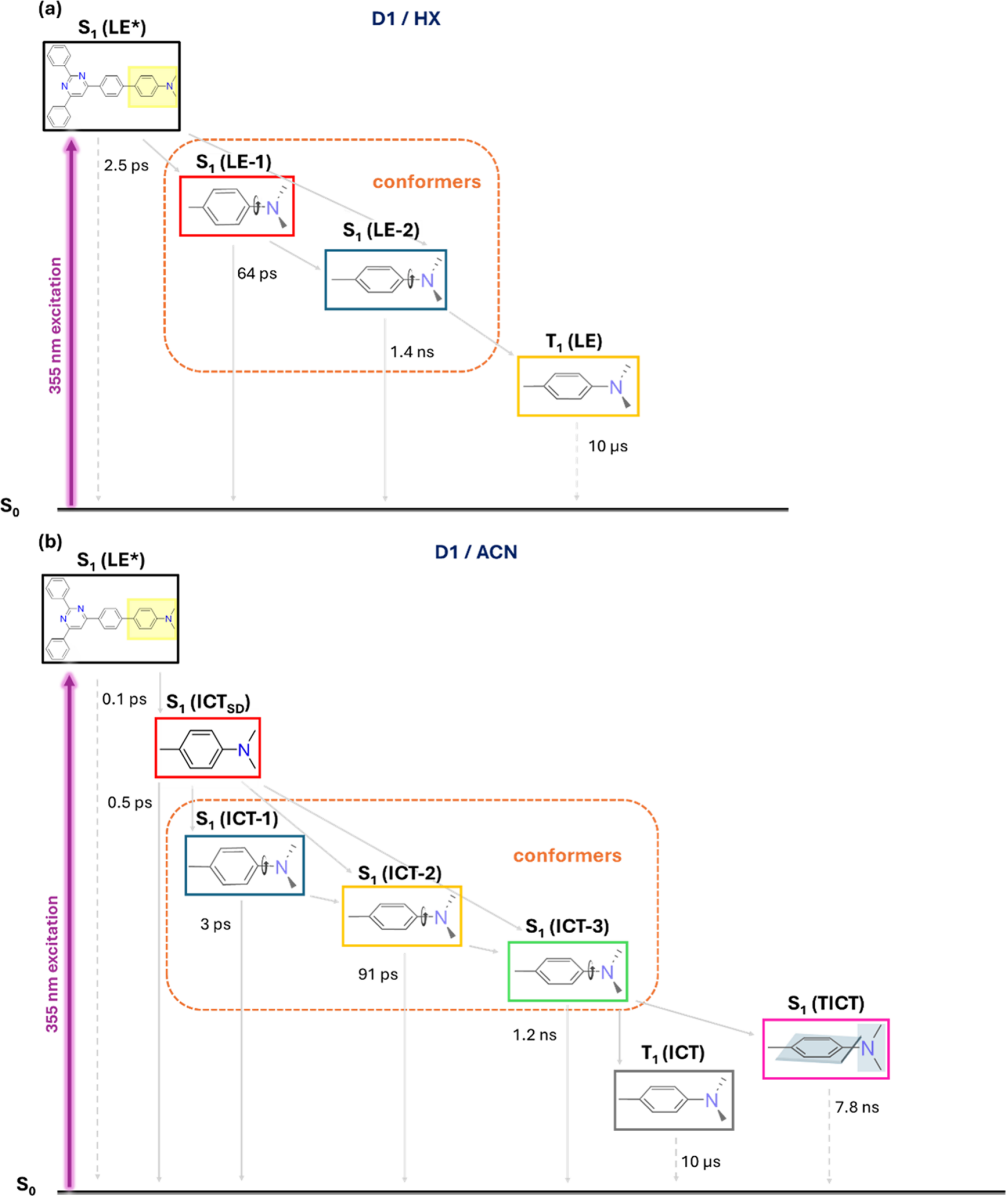
Schematic representation of the kinetic model used for target analysis of (a) **D1** in HX and (b) **D1** in ACN, indicating different excited-state species and corresponding lifetimes. For illustrative purposes only, the energy scale is not represented.^73^ The yellow highlighted region in S_1_ (LE*) is depicted in subsequent states for simplification.

Time-dependent DFT calculations from our previous work revealed that the C_5_C_6_C_7_C_8_ dihedral angles (corresponding to the biphenyl moiety; see Figure S26) in **D1** and **D2** become only slightly more planar in the excited state compared to the ground state, despite the substantial variation observed in the C_9_C_10_N_1_C_11_ dihedral angle (associated with the dimethylamino and phenyl units). These results indicate that the biphenyl fragment plays a minor role in the excited-state relaxation and therefore does not govern the overall photophysical behavior. On this basis, only the dihedral angles of **D1** corresponding to the dimethylamino and phenyl units, which undergo the most significant structural changes occur between the ground and excited states, are illustrated and discussed.

Fast photoinduced ICT of **D1** in ACN leads to an excited-state polarization in the molecule before solvent reorganization takes place. This is consistent with previous works on Nile blue or coumarins in electron-donating solvents, mostly anilines.^72^ Subsequently, the solvent molecules of ACN adapt their configurations toward the equilibrium of the excited state, or the so-called solvent dynamics. Since CT states are not expected in HX, the initial evolution of **D1** in HX reflects excess energy being dissipated through vibrational cooling, mediated by coupling between solute and solvent vibrational modes. Then, several conformers with distinct dihedral angles between different subunits are parallel- and sequentially populated (branch-type) from the previous state. It is noteworthy that the structures of S_1_ (LE-2) and S_1_ (ICT-3) for **D1** in HX and ACN, respectively, correspond to the planar conformation of S_1,0_ in our earlier study,^43^ where the dihedral angle between the biphenyl and *N*,*N*-dimethylamino terminus is 0, thus favoring electronic communication between the donor and acceptor moieties.

In highly polar solvents such as ACN and DMSO, the TICT state (S_1,90_) becomes now operative due to its lower energy and small activation energy barrier, which quenches the fluorescence from the ICT state. In our kinetic model, we propose that the triplet state is populated via the bright ICT state rather than directly from the dark TICT state, as the formation of triplet species is observed across all solvents, including those where the TICT state is either absent (HX and TOL).

Increasing the polarity a bit further from HX to TOL, **D1** already shows a significant spectral evolution at early times (Figure S20). Target analysis of **D1** in TOL revealed a spectrum similar to that obtained in HX, which evolved to a very different spectrum in just 0.3 ps. Consequently, this strongly supports the transformation of S_1_ (LE*) to S_1_ (ICT_SD_) already at early times.^74^ Further delay times (4.6 ps) change the shape around 540 nm with the disappearance of the shoulder around 480 nm and a narrowing of the ESA band through solvent reorganization dynamics. This S_1_ (ICT_SD_) state was connected to two conformers, namely S_1_ (ICT-1) and S_1_ (ICT-2), wherein the first one was longer-lived than that in HX, explaining that viscosity clearly exerts a strong effect on the twisting motion within the molecule. A similar time constant has been found for a decay of a relaxed LE state via twisting of the C=C bond in toluene.^75^ In contrast to the highly polar solvents, where TICT structures dominate, the TA spectra in nonpolar environments, such as TOL, are indicative of more planar CT states, reflecting substantially restricted conformational changes.^49^

For **D1** in DMSO, a similar kinetic fashion to that seen in ACN is encountered here (Figure S21).^76^ In principle, the same number of components was identified; but their evolution was slower than in ACN,^75^ primarily due to its higher solvent viscosity (1.99 cP vs 0.34 cP).^67^ Notably, the S_1_ (TICT) state in DMSO readily forms and exhibits a shorter lifetime (2.0 ns) compared to that in ACN (7.8 ns), as a consequence of the increased solvent polarity of the former (*E*_T_(30) = 45.1 vs 37.5),^77^ facilitating even faster nonradiative deactivation of the S_1_ (ICT-3) state. Nevertheless, one of the intermediate ICT states, S_1_ (ICT-2), appears to persist even longer, again reflecting a rotational process in which solvent viscosity plays a dominant role, despite the increased polarity of DMSO.

The marked decrease in fluorescence quantum yield and excited-state lifetime of the ICT emission band with increasing solvent polarity, together with the emergence of TICT-related species in the fs-TAS analysis, strongly support the formation of a TICT state in these push–pull systems. The observed viscosity dependence further corroborates that intramolecular twisting motion governs the relaxation dynamics.

**D2** behaves similarly to **D1** in nonpolar solvents, with only minimal differences in dynamics (Figures S22 and S23). Thus, **D1** exhibits faster excited-state relaxation dynamics than **D2**. For instance, motion-related states are slightly prolonged in **D2**, likely due to the bulky substituents on the donor moiety. However, in highly polar solvents, very long-lived S_1_ (ICT-3) were detected (Figures S24 and S25), in accordance with TCSPC measurements,^78^ where the relaxation of the S_1_ (ICT-3) state in **D2** is directly through the radiative and nonradiative transitions to the ground state, being the primary pathway of decreasing the fluorescence quantum yield.

All lifetimes obtained through target analysis are tabulated in Table S4. Interestingly, the lifetimes of S_1_ (ICT-2) and S_1_ (ICT-3) correlate well with both viscosity and dielectric constant for both **D1** and **D2** compounds, as observed in Figure S28. An increase in solvent viscosity leads to longer-lived lifetimes of the S_1_ (ICT-2) state for both (although better correlation for **D2**) whereas S_1_ (ICT-3) becomes gradually shortened and lengthened for **D1** and **D2**, respectively, with increasing dielectric constant. These trends are fully consistent with our previous Φ_F_ measurements.^43^

Although we previously assigned the ICT absorption bands to transitions from the ground state,^43^ the emission mapping and transient absorption data presented here confirm that the initially populated excited state is the locally excited (LE) state (or at least involves partial population of it). This discrepancy can be explained by the use of B3LYP functionals in the former work, which are known to underestimate the ICT energy.^79^

Particularly, **D1** and **D2** push–pull molecules are intrinsically complex systems, characterized by multiple electronic states and structural flexibility involving numerous atoms, bonds, and dihedral angles, as evidenced by TA results. This complexity can significantly influence their photophysical behavior and complicate theoretical modeling. However, our proposed model is fully consistent with the experimental results shown above. Additionally, it was able to resolve the TICT spectrum in both cases (**D1** in ACN and DMSO), despite the large number of components involved in the analysis. Moreover, the TICT state population was proposed to be directly formed through a unique pathway, namely, the solvated ICT state with full structural relaxation.

In a nutshell, these findings highlight the strong influence of both solvent polarity and viscosity on the population, lifetime, and evolution of emissive states in complex push–pull systems, particularly through solvent-stabilized charge–transfer pathways and conformer-specific dynamics. Undoubtedly, gaining deeper insight into the nature and energetics of the excited states, particularly the subtle interplay between LE, ICT, TICT, and triplet pathways, would benefit from quantum-chemical theoretical calculations. However, due to the structural complexity of the studied systems, involving numerous atoms, bonds, and flexible dihedral angles, such computational studies fall beyond the scope of the present work. Future theoretical efforts focusing on excited-state potential energy surfaces and conformational dynamics would be invaluable for complementing and validating the experimental observations reported here.

## 4 Conclusions

In this study, novel insights into the complex landscape of the excited state in these push–pull systems have been gained through a combination of steady-state (emission mapping), time-correlated single photon counting (TCSPC), and transient absorption (fs/ns-TAS) measurements. First, the emergence of dual emission for both systems in all solvents (except for hexane) proves the population of at least two emitting states, namely locally excited (LE) and intramolecular charge transfer (ICT) states. Next, a closer examination of the time-resolved fluorescence using TCSPC showcases a precursor–successor relationship between the LE and ICT bands, where the ICT band of **D1** is significantly quenched in highly polar solvents, while **D2** maintains its ICT emission under similar conditions. Thus, conformational effects have a significant photophysical impact, as the diphenylamino group in **D2** experiences restricted rotation compared to the more flexible dimethylamino group in **D1**. More importantly, ultrafast spectroscopic investigations revealed the coexistence of multiple excited-state species associated with different conformers, whose lifetimes display a pronounced dependence on solvent viscosity. Collectively, these species contribute to a complex excited-state deactivation cascade, shaped by both molecular flexibility and environmental factors. While the ICT-3 state of **D1** shortens with increasing solvent polarity, this behavior contrasts with that of **D2**, where the absence of a TICT state results in this specie becoming more long-lived, aligning with our previous findings and those obtained from TCSPC measurements. Therefore, the results of this new study profoundly highlight the critical influence of both solvent and molecular conformation on the excited-state deactivation pathways of push–pull systems. These insights not only deepen our fundamental understanding of their photophysics but also provide a valuable framework for the rational design of next-generation functional materials with tailored optical and electronic properties.

## Supplementary Material



## Notes

Steady-state absorption and emission properties are presented alongside emission mapping and TCSPC measurements in various solvents (Figures S1–S5, Table S1). TRES maps (Figures S6 and S7) and transient absorption spectroscopy spanning femtosecond to nanosecond time scales detail solvent-dependent excited-state dynamics (Figures S8–S15, Tables S2 and S3). Additional studies include spectroelectrochemistry, phosphorescence, and singlet oxygen emission (Figures S16–S19). Global and target analyses with corresponding species-associated difference spectra (SADS) are shown for both systems in multiple solvents (Figures S20–S25). Finally, optimized ground- and excited-state geometries (Figures S26 and S27) and correlations of fs-TA time constants with solvent viscosity and dielectric constant (Figure S28) are provided (PDF)
