# Supplementary material for: Excited-State Relaxation Pathways of 4‑Aminobiphenyl-2-Pyrimidine Derivatives: An Ultrafast Perspective
Source: J Phys Chem A. 2025 Oct 31;129(45):10516–28. doi: 10.1021/acs.jpca.5c06504 (PMC12621238; doi:10.1021/acs.jpca.5c06504)
Supplement: Supplementary file 1 [file jp5c06504_si_001.pdf]

Supplementary Information

Excited-State Relaxation Pathways of 4-Aminobiphenyl-2-Pyrimidine Derivatives: An Ultrafast Perspective

Alejandro Cortés-Villena[1,2], Soranyel Gonzalez-Carrero[1,2], Carolina Aliaga[3]*, Moisés Domínguez[3], Matías Vidal[3], Pablo Rojas[3], Raquel E. Galian[1]* and Julia Pérez-Prieto[1]**

[1]Instituto de Ciencia Molecular, Universidad de Valencia, Valencia, Spain.

[2]Department of Chemistry and Centre for Processable Electronics, Imperial College London, London, United Kingdom.

[3]Facultad de Química y Biología, Universidad de Santiago de Chile, Santiago, Chile.

Table of Contents

Figure S1. Steady-state optical properties. S3

Figure S2. Viscosity-dependent fluorescence. S3

Figure S3. Emission mapping and TCSPC of **D1** in TOL and DMSO. S4

Figure S4. Emission mapping and TCSPC of **D2** in HX and TOL. S4

Figure S5. Emission mapping and TCSPC of **D2** in ACN and DMSO. S5

Table S1. Time constants from TCSPC. S5

Figure S6. TRES map for **D1**. S7

Figure S7. TRES map for **D2**. S7

Figure S8. fs-TA spectra and kinetics for **D1** in TOL and DMSO. S8

Figure S9. fs-TA spectra and kinetics for **D2** in HX and TOL. S8

Figure S10. fs-TA spectra and kinetics for **D2** in ACN and DMSO. S9

Figure S11. ns-TA spectra and kinetics for **D1** in HX and TOL. S9

Figure S12. ns-TA spectra and kinetics for **D1** in ACN and DMSO. S10

Figure S13. ns-TA spectra and kinetics for **D2** in HX and TOL. S10

Figure S14. ns-TA spectra and kinetics for **D2** in ACN and DMSO. S11

Table S2. Time constants from ns-TAS. S12

Figure S15. ns-TA kinetics of **D1** in HX under N_2 and O_2 conditions. S12

Figure S16. SEC of **D2** in ACN. S13

Figure S17. Phosphorescence spectra. S13

Figure S18. $^1O_2$ phosphorescence spectra of **D1**. S14

Figure S19. $^1O_2$ phosphorescence spectra of **D2**. S15

Figure S20. SADS for **D1** in TOL. S16

Figure S21. SADS for **D1** in DMSO. S16

Figure S22. SADS for **D2** in HX. S17

Figure S23. SADS for **D2** in TOL. S18

Figure S24. SADS for **D2** in ACN. S18

Figure S25. SADS for **D2** in DMSO. S19

Table S3. Time constants from target analysis. S19

Figure S26. Optimized geometries for the ground state. S20

Figure S27. Optimized geometries for the first excited state. S21
Figure S28. Relationship of fs-TA time constants with viscosity and dielectric constant. S22

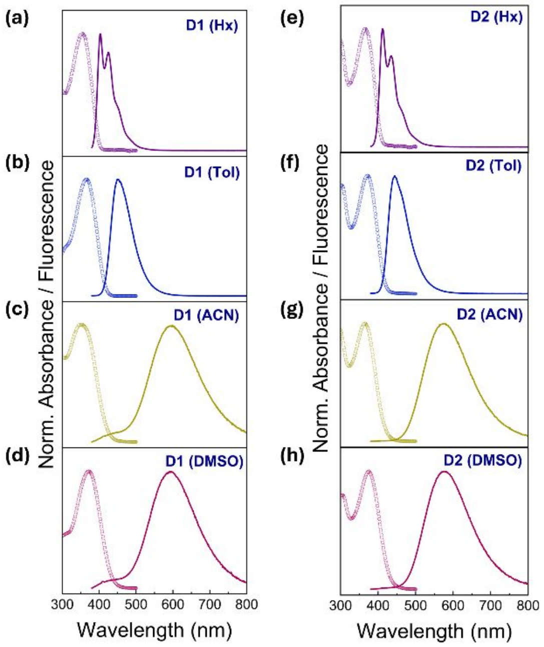

Figure S1. Steady-state absorption and fluorescence spectra of **D1** in HX (a), TOL (b), ACN (c), and DMSO (d). (e-h) the show the corresponding data for **D2**.

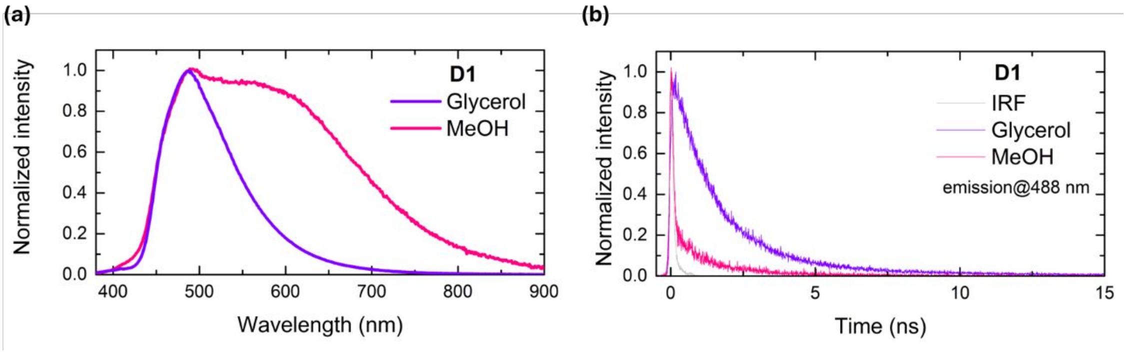

Figure S2. (a) Normalized fluorescence spectra and (b) normalized kinetic traces at 488 nm for D1 in Glycerol (purple) and MeOH (pink). Continuous-wave excitation at 365 nm for (a) and pulsed-laser excitation at 375 nm for (b), respectively.

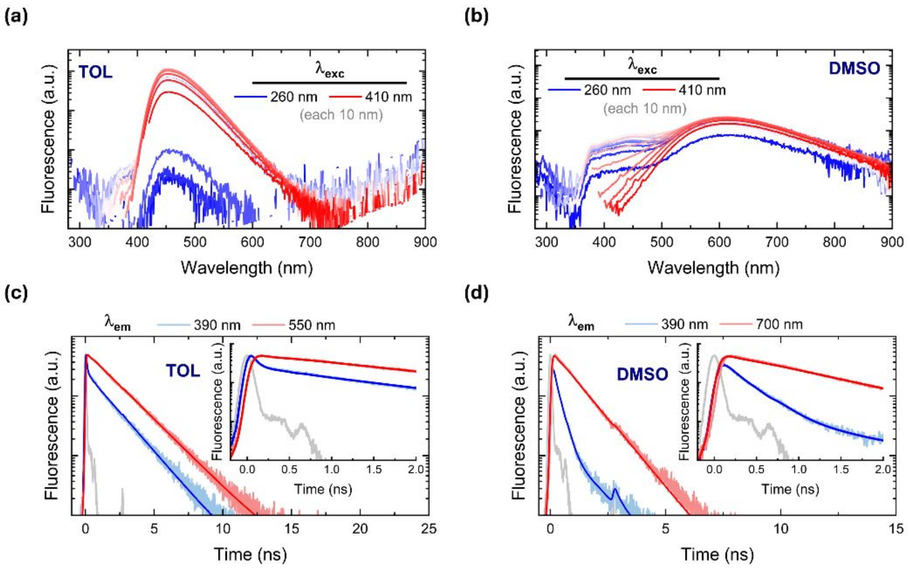

Figure S3. (a) Steady-state fluorescence spectra of (a) **D1** in TOL and (b) **D1** in DMSO under varying excitation wavelengths (260-410 nm; each 10 nm). Fluorescence in log scale. Fluorescence decay kinetics for (c) **D1** in TOL at 390 and 550 nm and for (d) **D1** in DMSO at 390 and 700 nm under 375 nm picosecond pulsed-laser excitation (inset: early-time trace).

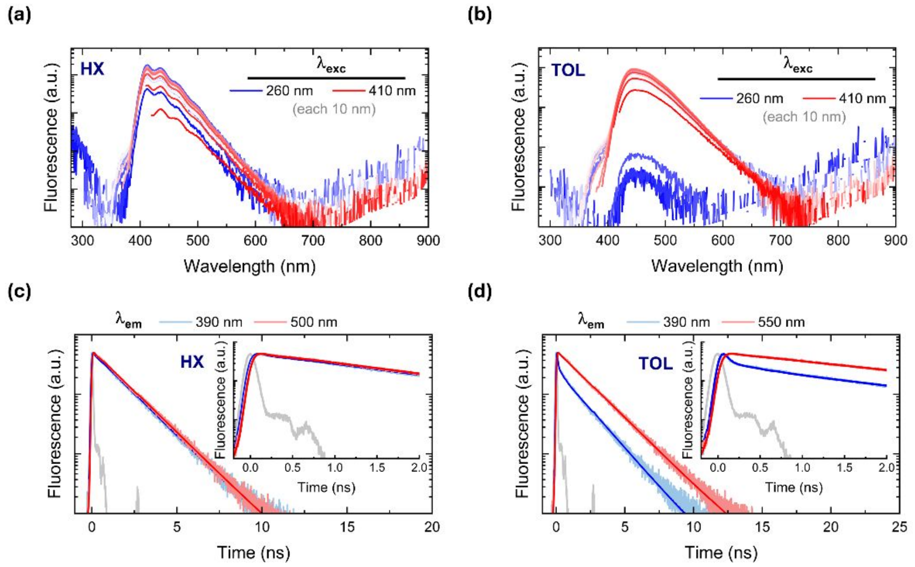

Figure S4. (a) Steady-state fluorescence spectra of (a) **D2** in HX and (b) **D2** in TOL under varying excitation wavelengths (260-410 nm; each 10 nm). Fluorescence in log scale. Fluorescence decay kinetics for (c) **D2** in HX at 390 and 500 nm and for (d) **D2** in TOL

at 390 and 550 nm under 375 nm picosecond pulsed-laser excitation (inset: early-time trace).

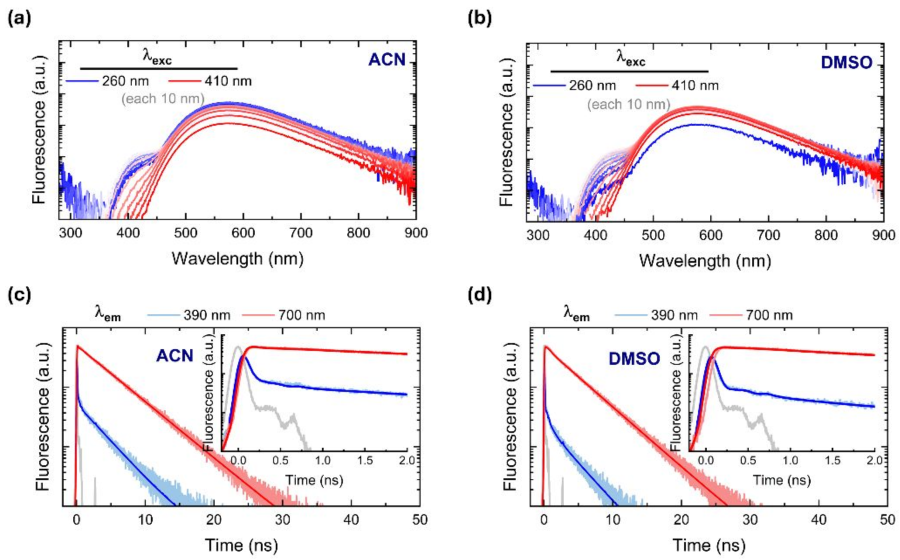

Figure S5. (a) Steady-state fluorescence spectra of (a) **D2** in ACN and (b) **D2** in DMSO under varying excitation wavelengths (260-410 nm; each 10 nm). Fluorescence in log scale. Fluorescence decay kinetics for (c) **D2** in ACN at 390 and 700 nm and for (d) **D2** in DMSO at 390 and 700 nm under 375 nm picosecond pulsed-laser excitation (inset: early-time trace).

Table S1. Fitting parameters obtained from TCSPC data through multiexponential fitting for **D1** and **D2** in different solvents. A 375 nm picosecond pulsed-laser excitation source was used (10 MHz). The instrumental response function (IRF) is around 120 ±10 ps.

Compound	Solvent	Emission	τ_1 (ps)	B_1 (%)	τ_2 (ps)	B_2 (%)	τ_3 ± sd (ps)	B_3 (%)	τ_{av} ± sd (ps)	χ^2
D1	HX	390 nm			64*	2	1359 ±1	98	1335 ±3	1.18

		**500 nm**			64*	1	1501 ±1	99	1480 ±3	1.17
	**TOL**	**390 nm**	3*	8	122*	5	163 ±3	87	1428 ±218	1.58
		**550 nm**			122*	1	1898 ±2	99	1875 ±2	1.08
	**ACN**	**390 nm**	3*	26	91*	10	1175 ±6	64	763 ±407	1.53
		**700 nm**			91*	2	1312 ±2	98	1291 ±3	1.22
	**DMSO**	**390 nm**			145*	83	1043 ±17	17	343 ±6	1.46
		**700 nm**			145*	2	920 ±1	98	902 ±2	3.45
**D2**	**HX**	**390 nm**			106*	3	1530 ±2	97	1484 ±3	1.29
		**500 nm**			106*	2	1556 ±2	98	1525 ±2	1.20
	**TOL**	**390 nm**	3*	7	196*	7	1654 ±3	86	1432 ±211	1.46
		**550 nm**			196*	3	1920 ±2	97	1876 ±2	1.14
	**ACN**	**390 nm**	5*	14	133*	5	3439 ±9	81	2788 ±534	1.29
		**700 nm**			133*	1	4573 ±3	99	4529 ±3	1.72
	**DMSO**	**390 nm**	18*	30	700*	11	3357 ±20	59	2076 ±208	1.21
		**700 nm**			700*	3	4291 ±3	97	4183 ±4	6.92

Fixed values from fs-TAS are indicated with an asterisk (*). Standard deviation (sd) is shown for τ_3 and τ_{av}.

The data is fitted to a multiexponential decay function:

$$I(\mathrm{t}) = B_1 e^{\left(-t/\tau_1\right)} + B_2 e^{\left(-t/\tau_2\right)} + B_3 e^{\left(-t/\tau_3\right)} \qquad \text{(S1)}$$

with τ_i and B_i are the lifetime and amplitude, respectively as fitting parameters of the *ith* exponential component.

The intensity average lifetime value is calculated according to: $\tau_{av} = \frac{\sum_{i=1}^{n} B_i \tau_i^2}{\sum_{i=1}^{n} B_i \tau_i}$ (S2)

The relative emission intensity is calculated as follows: $B_1(\%) = \frac{B_1 \tau_1}{\sum_{i=1}^{n} B_i \tau_i} x100$ (S3)

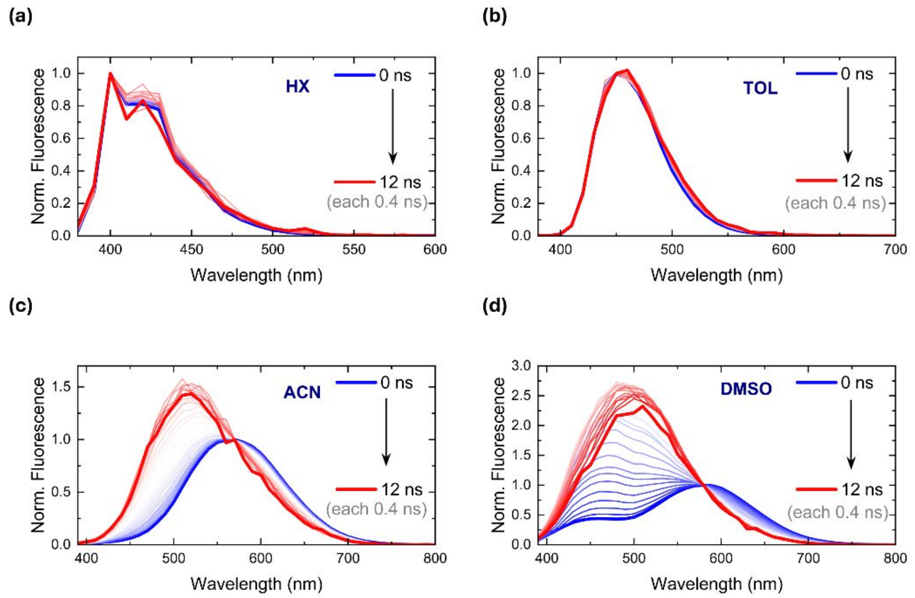

Figure S6. Normalized time-resolved emission spectra for **D1** in HX (a), TOL (b), ACN (c) and DMSO (d).

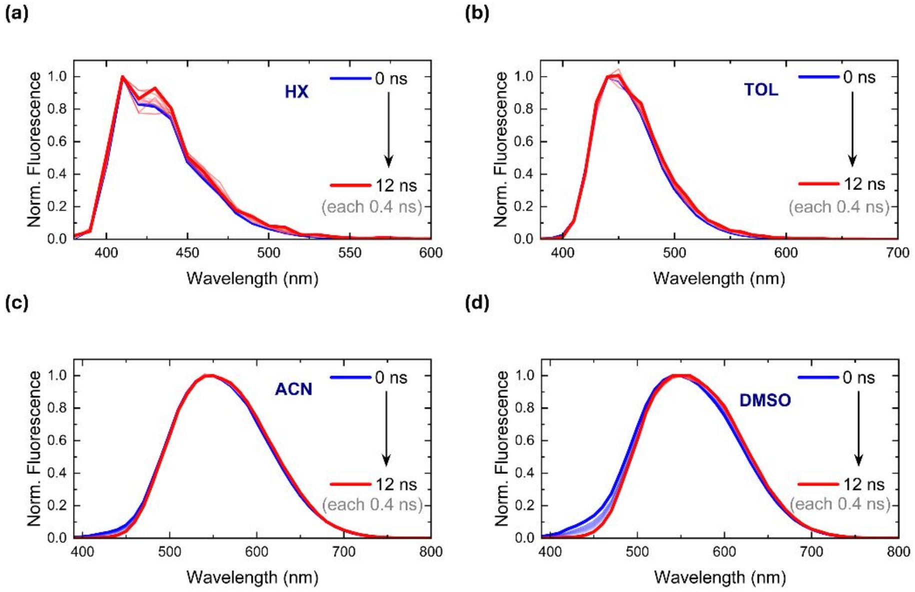

Figure S7. Normalized time-resolved emission spectra for **D2** in HX (a), TOL (b), ACN (c) and DMSO (d).

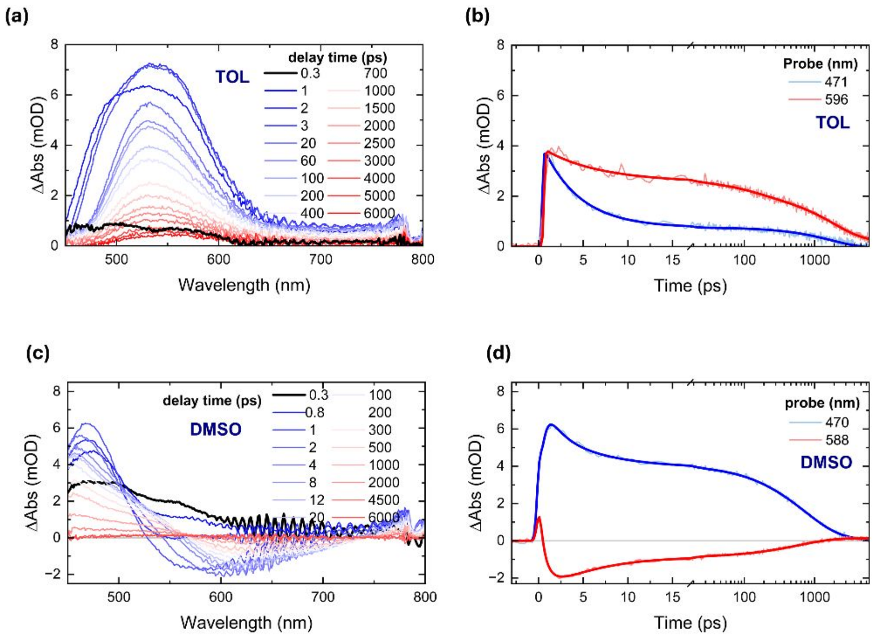

Figure S8. (a) fs-TA spectra of **D1** in TOL (λ_{pump} = 355 nm) at indicated delay times and (b) two kinetic traces at two representative wavelengths. (c) fs-TA spectra of **D1** in DMSO (λ_{pump} = 355 nm) at indicated delay times and (d) two kinetic traces at two representative wavelengths. Exponential fittings are included (solid lines) in all kinetic traces.

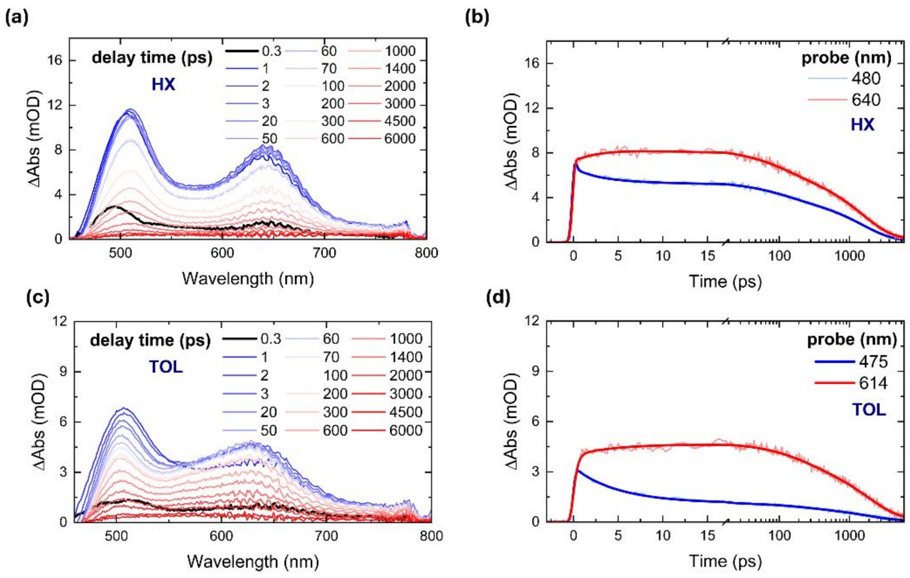

Figure S9. (a) fs-TA spectra of **D2** in HX (λ_{pump} = 355 nm) at indicated delay times and (b) two kinetic traces at two representative wavelengths. (c) fs-TA spectra of **D2** in TOL (λ_{pump} = 355 nm) at indicated delay times and (d) two kinetic traces at two representative wavelengths. Exponential fittings are included (solid lines) in all kinetic traces.

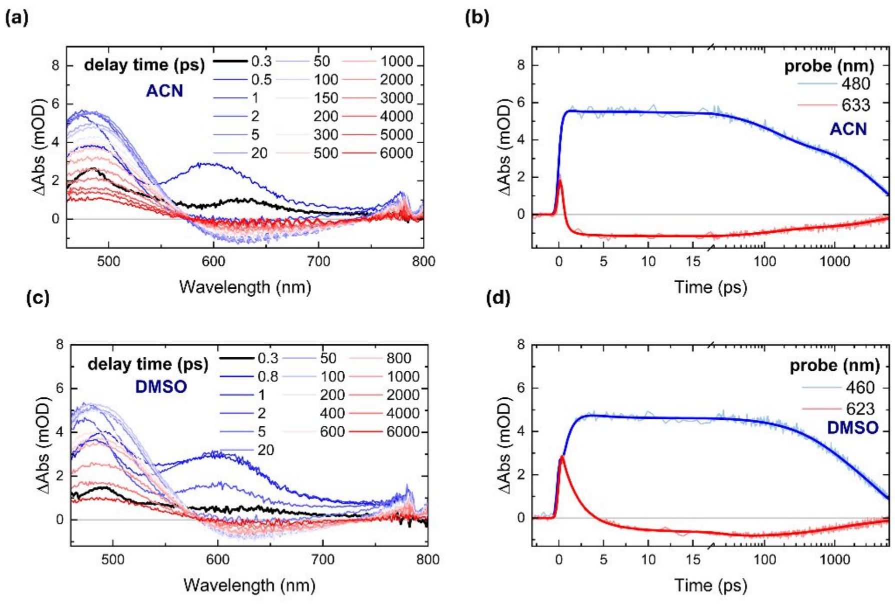

Figure S10. (a) fs-TA spectra of **D2** in ACN (λ_{pump} = 355 nm) at indicated delay times and (b) two kinetic traces at two representative wavelengths. (c) fs-TA spectra of **D2** in

DMSO (λ_{pump} = 355 nm) at indicated delay times and (d) two kinetic traces at two representative wavelengths. Exponential fittings are included (solid lines) in all kinetic traces.

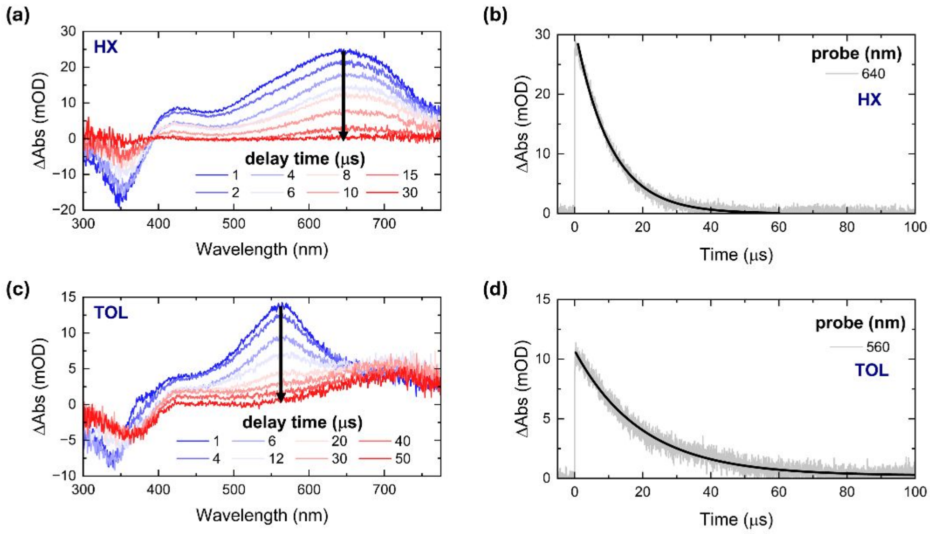

Figure S11. (a) ns-TA spectra of **D1** in HX (λ_{pump} = 355 nm) at indicated delay times and (b) kinetic trace at 640 nm. (c) ns-TA spectra of **D1** in TOL (λ_{pump} = 355 nm) at indicated delay times and (d) kinetic trace at 560 nm. Black lines indicate single exponential fitting.

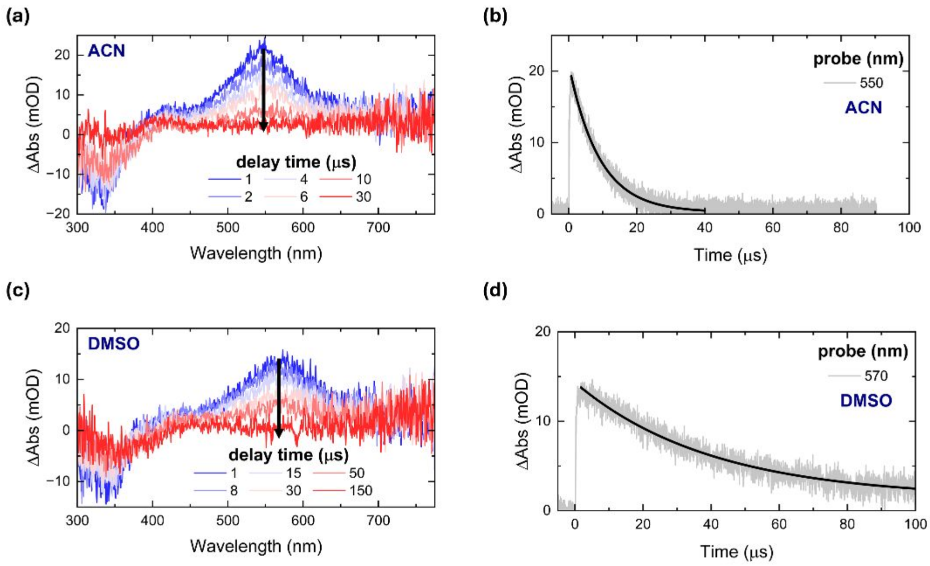

Figure S12. (a) ns-TA spectra of **D1** in ACN (λ_{pump} = 355 nm) at indicated delay times and (b) kinetic trace at 550 nm. (c) ns-TA spectra of **D1** in DMSO (λ_{pump} = 355 nm) at indicated delay times and (d) kinetic trace at 570 nm. Black lines indicate single exponential fitting.

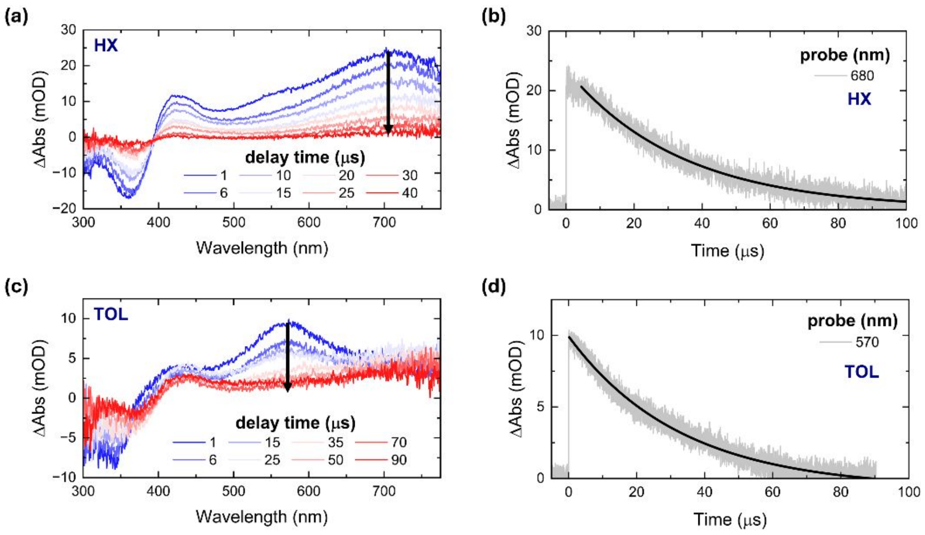

Figure S13. (a) ns-TA spectra of **D2** in HX (λ_{pump} = 355 nm) at indicated delay times and (b) kinetic trace at 680 nm. (c) ns-TA spectra of **D2** in TOL (λ_{pump} = 355 nm) at indicated delay times and (d) kinetic trace at 570 nm. Black lines indicate single exponential fitting.

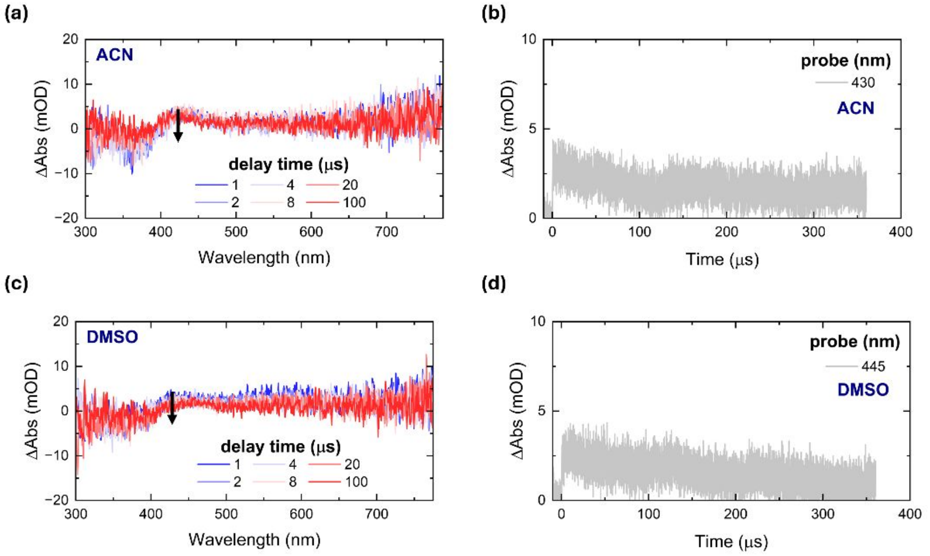

Figure S14. (a) ns-TA spectra of **D2** in ACN (λ_{pump} = 355 nm) at indicated delay times and (b) kinetic trace at 430 nm. (c) ns-TA spectra of **D2** in DMSO (λ_{pump} = 355 nm) at indicated delay times and (d) kinetic trace at 445 nm.

Table S2. Fitting parameters obtained from single exponential fitting of the kinetic traces for **D1** and **D2** in different solvents. A 355 nm pump excitation with 5 mJ/pulse was used in all of them. The instrumental response function (IRF) is around 10 ±2 ns.

Compound	Solvent	Probe	τ (μs)
D1	HX	640 nm	10.35 ±0.04
	TOL	560 nm	19.7 ±0.1
	ACN	550 nm	8.97 ±0.06
	DMSO	570 nm	40.0 ±0.6
D2	HX	680 nm	33.6 ±0.3
	TOL	570 nm	32.9 ±0.2
	ACN	-	-
	DMSO	-	-

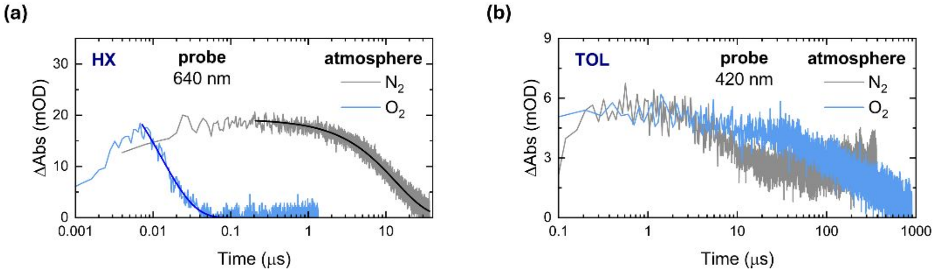

Figure S15. (a) Kinetic trace of **D1** in HX from ns-TAS (λ_{pump} = 355 nm) probed at 640 nm under nitrogen- and oxygen-saturated conditions. (b) Kinetic trace of **D1** in TOL from ns-TAS (λ_{pump} = 355 nm) probed at 420 nm under nitrogen- and oxygen-saturated conditions. Time axis are in logarithmic scale for both.

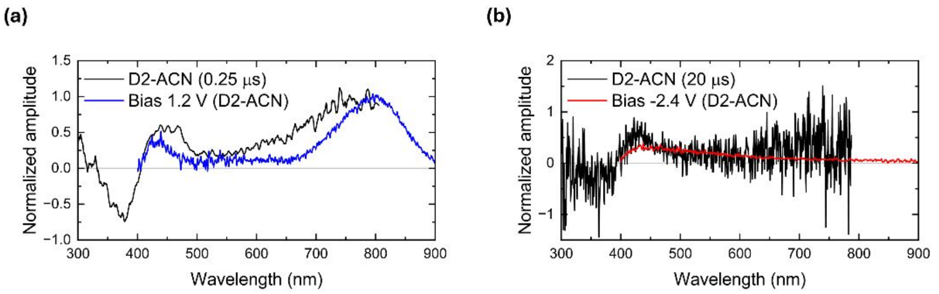

Figure S16. (a) Comparison of the extracted ns-TA spectrum at indicated delay time for **D2** in ACN with those obtained spectroelectrochemically under (a) positive (1.2 V vs. Ag/AgCl) and (b) negative bias (-2.4 V vs. Ag/AgCl), respectively.

(a)

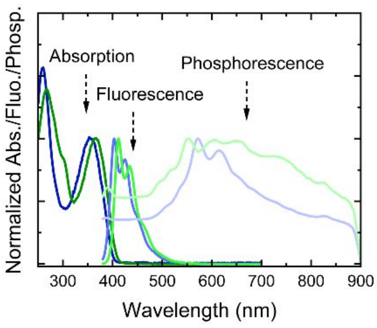

(b)

Sample	T= -196ºC	Phosphorescence max. (nm)	Energy of Triplet T_1 (eV)
D1	Solid matrix-hexane	572	2.17
D2	Solid matrix-hexane	552	2.25

Figure S17. (a) Normalized absorption, fluorescence and phosphorescence spectra of **D1** (blue) and **D2** (green). Absorption and fluorescence are in HX while phosphorescence is in HX under nitrogen liquid conditions. The excitation wavelength was 365 nm. (b) Table summarizing measurement conditions, phosphorescence maxima and triplet energy.

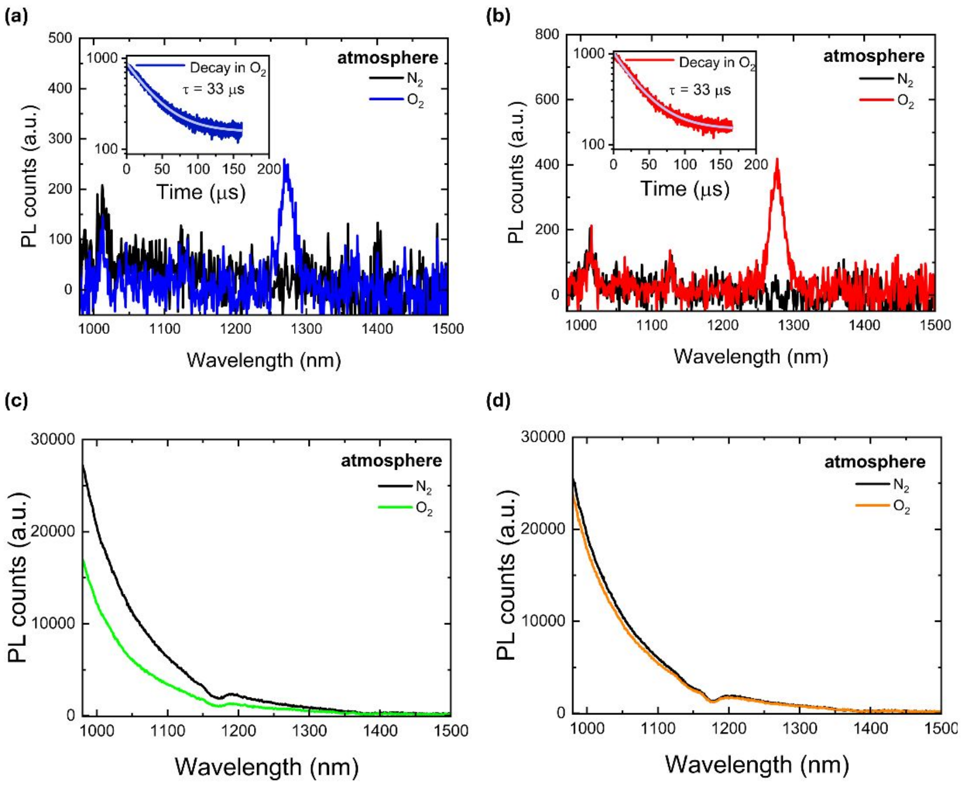

Figure S18. $^1O_2$ phosphorescence spectra photosensitized by **D1** measured under nitrogen- and oxygen-saturated conditions in HX (a), TOL (b), ACN (c), and DMSO (d). The excitation wavelength was 365 nm. Inset of (a) and (b) shows the kinetic trace for $^1O_2$ at 1270 nm in those solvents.

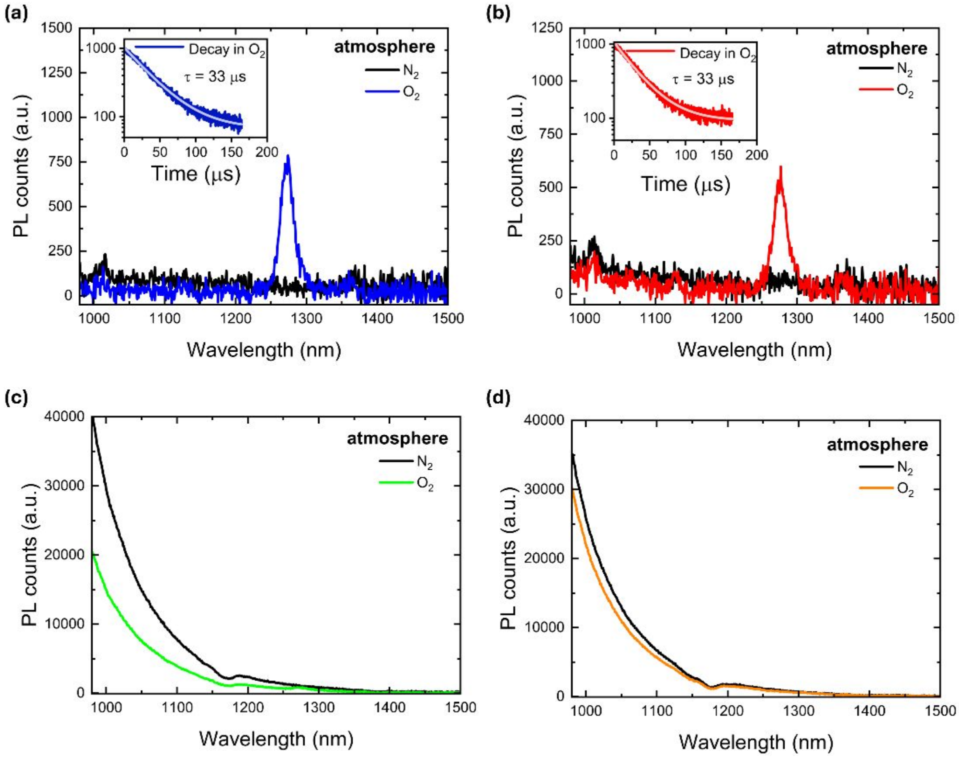

Figure S19. ${}^1O_2$ phosphorescence spectra photosensitized by **D2** measured under nitrogen- and oxygen-saturated conditions in HX (a), TOL (b), ACN (c), and DMSO (d). The excitation wavelength was 365 nm. Inset of (a) and (b) shows the kinetic trace for ${}^1O_2$ at 1270 nm in those solvents.

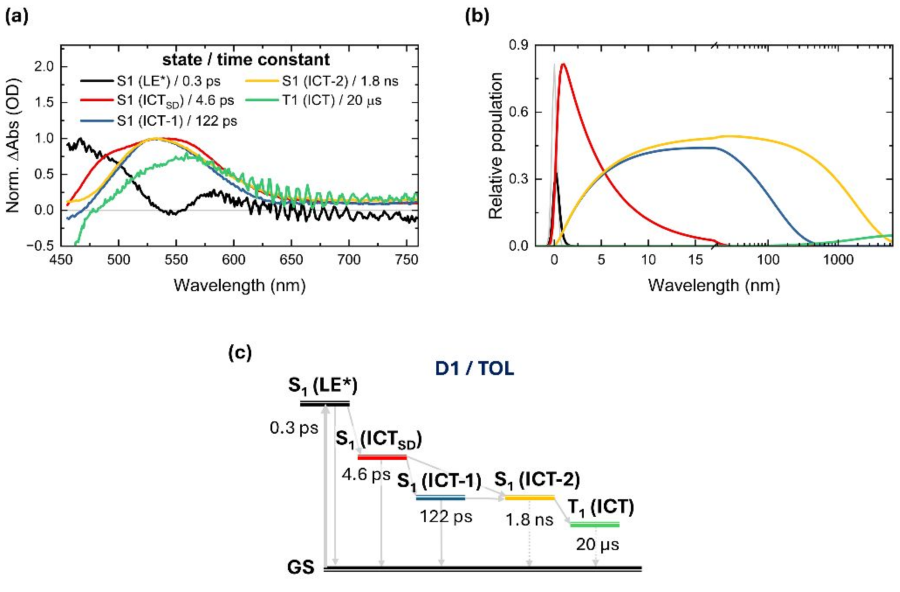

Figure S20. (a) SADS of **D1** in TOL along with the time constants obtained through global target analysis and (b) their corresponding population profile over time. (c) Kinetic model used for target analysis of **D1** in TOL. For illustrative purposes only; energy scale is not represented.

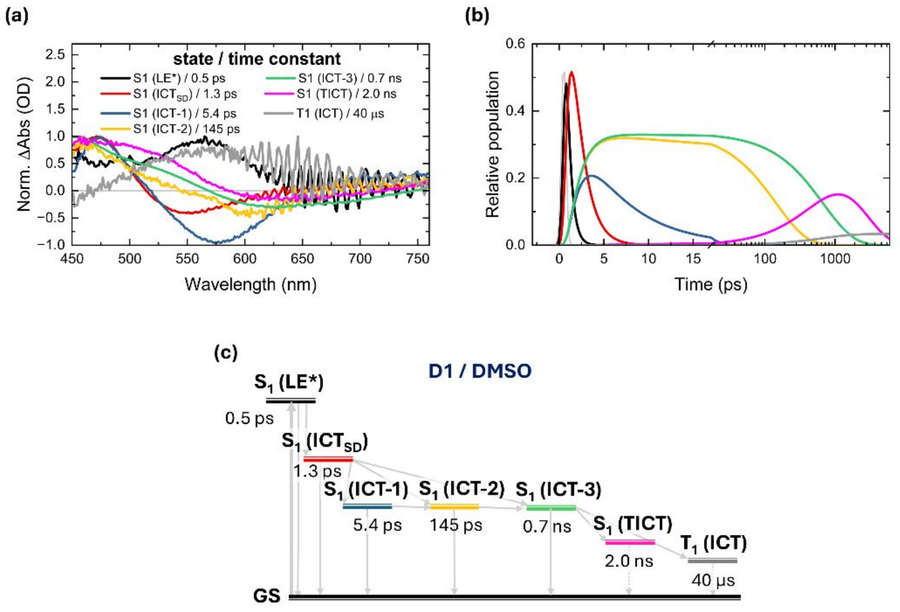

Figure S21. (a) SADS of **D1** in DMSO along with the time constants obtained through global target analysis and (b) their corresponding population profile over time. (c) Kinetic

model used for target analysis of **D1** in DMSO. For illustrative purposes only; energy scale is not represented.

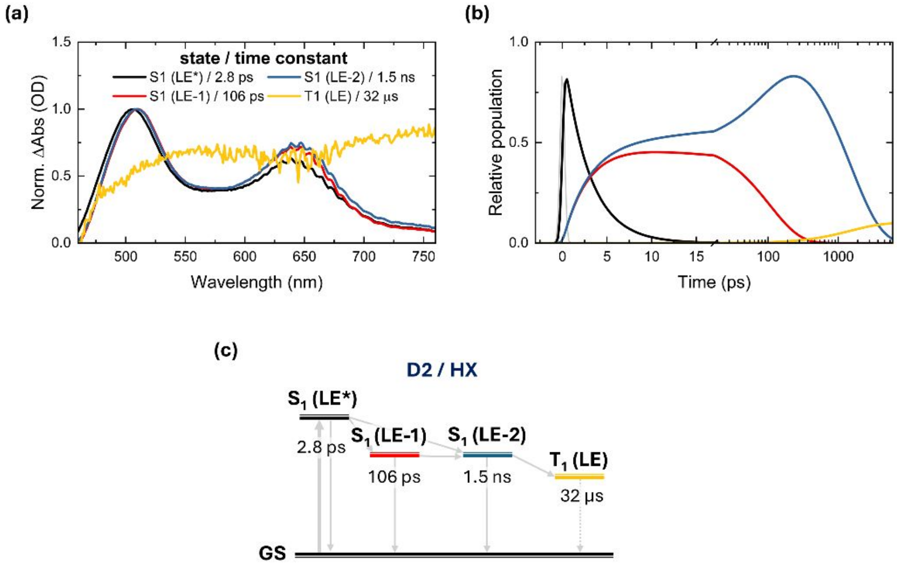

Figure S22. (a) SADS of **D2** in HX along with the time constants obtained through global target analysis and (b) their corresponding population profile over time. (c) Kinetic model used for target analysis of **D2** in HX. For illustrative purposes only; energy scale is not represented.

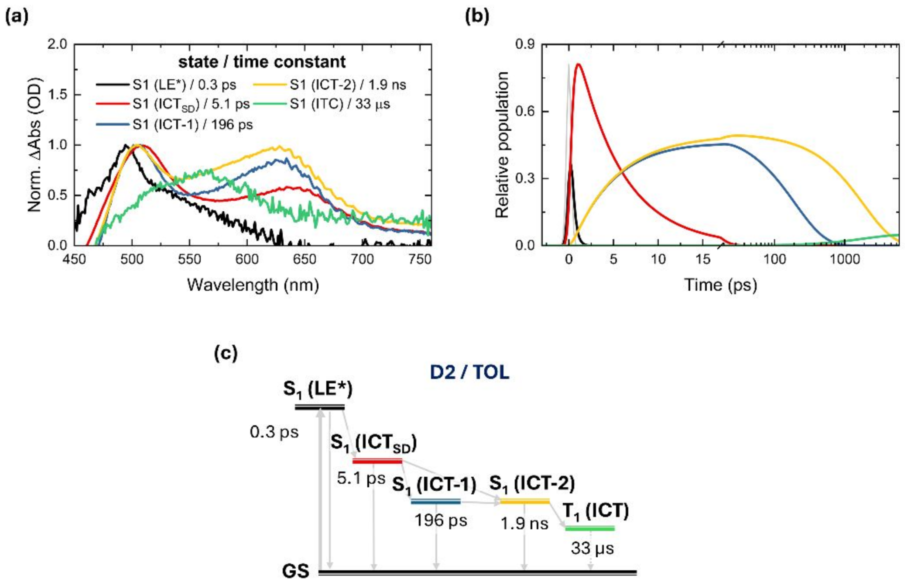

Figure S23. (a) SADS of **D2** in TOL along with the time constants obtained through global target analysis and (b) their corresponding population profile over time. (c) Kinetic model used for target analysis of **D2** in TOL. For illustrative purposes only; energy scale is not represented.

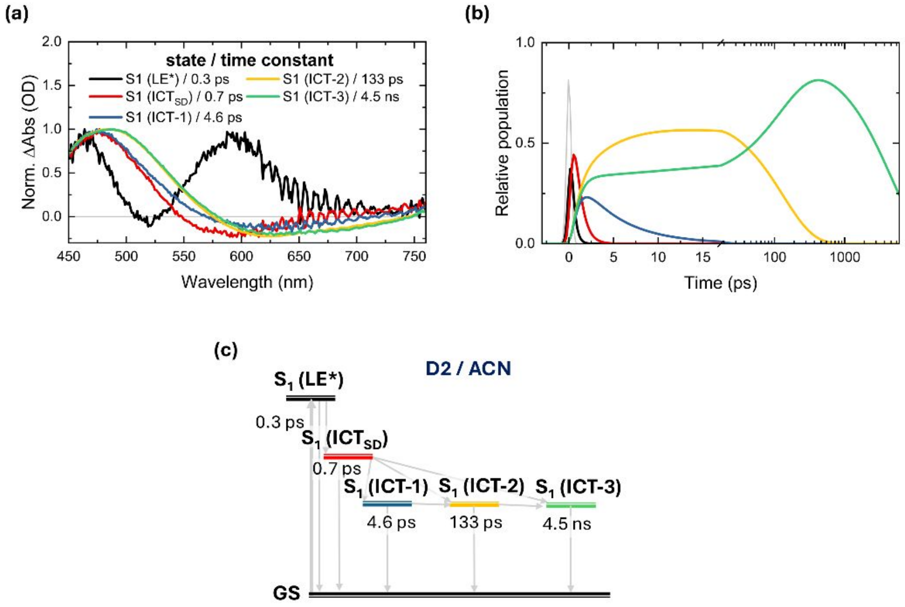

Figure S24. (a) SADS of **D2** in ACN along with the time constants obtained through global target analysis and (b) their corresponding population profile over time. (c) Kinetic

model used for target analysis of **D2** in ACN. For illustrative purposes only; energy scale is not represented.

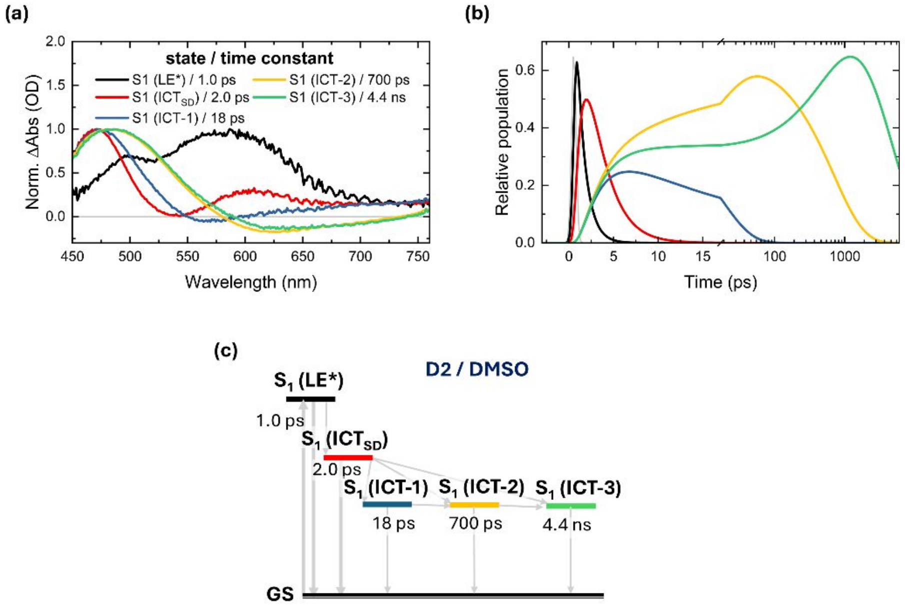

Figure S25. (a) SADS of **D2** in DMSO along with the time constants obtained through global target analysis and (b) their corresponding population profile over time. (c) Kinetic model used for target analysis of **D2** in DMSO. For illustrative purposes only; energy scale is not represented.

Table S3. Fitting parameters obtained from global target analysis of the transient absorption matrix for **D1** and **D2** in different solvents. A 355 nm pump excitation with 100 nJ/pulse was used in all of them. The instrumental response function (IRF) is around 0.25 ps.

Compound	Solvent	τ_1 (ps)	τ_2 (ps)	τ_3 (ps)	τ_4 (ps)	τ_5 (ps)	τ_6 (ps)	τ_7 (ps)
D1	HX	2.5 ±0.1	63.5 ±0.2	1425 ±1	10000000*			

	TOL	0.27 ±0.01	4.56 ±0.01	122 ±1	1819 ±4	20000000*		
	ACN	0.1*	0.5*	3*	91 ±1	1227 ±9	7760 ±2330	20000000*
	DMSO	0.5±0.1	1.28 ±0.02	5.42 ±0.04	145 ±2	685 ±5	1952 ±438	40000000*
D2	HX	2.83 ±0.03	106.1 ±0.3	1524 ±2	32000000*			
	TOL	0.3*	5.06 ±0.02	196 ±1	1868 ±4	33000000*		
	ACN	0.3*	0.7 ±0.3	4.6 ±0.1	133 ±1	4499 ±4		
	DMSO	0.96 ±0.01	1.99 ±0.02	17.6 ±0.2	699 ±4	4396 ±10		

Fixed values are indicated with an asterisk (*). Standard deviation (sd) is also shown.

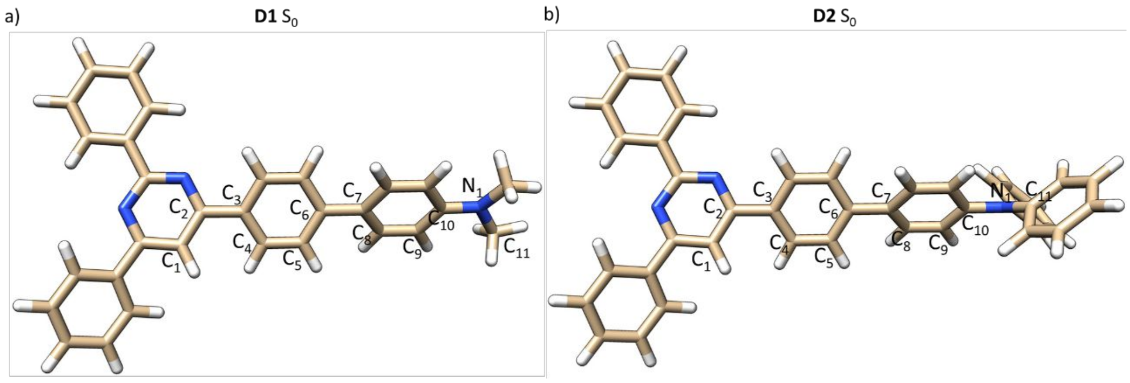

Solvent	Dihedral	D1	D2
HX	$C_1C_2C_3C_4$	21.35	21.81
	$C_5C_6C_7C_8$	34.35	35.65
	$C_9C_{10}N_1C_{11}$	8.59	37.59
TOL	$C_1C_2C_3C_4$	21.17	21.91
	$C_5C_6C_7C_8$	33.93	35.39
	$C_9C_{10}N_1C_{11}$	8.45	37.44
ACN	$C_1C_2C_3C_4$	20.81	22.60
	$C_5C_6C_7C_8$	31.37	34.16
	$C_9C_{10}N_1C_{11}$	-8.46	36.65
DMSO	$C_1C_2C_3C_4$	20.80	22.51
	$C_5C_6C_7C_8$	31.34	34.12
	$C_9C_{10}N_1C_{11}$	-8.46	36.59

Figure S26. DFT/B3LYP/6-311G**-optimized geometries calculated for the ground state (S_0) of **D1** (a) and **D2** (b). The values of the dihedral angles defining the optimized structure are given in the table expressed in degrees. Reproduced from reference [1]. Copyright © 2023 Cortés-Villena, Soriano-Díaz, Domínguez, Vidal, Rojas, Aliaga,

Giussani, Doménech-Carbó, Ortí, Galian and Pérez-Prieto. This is an open-access article distributed under the terms of the Creative Commons Attribution License (CC BY).

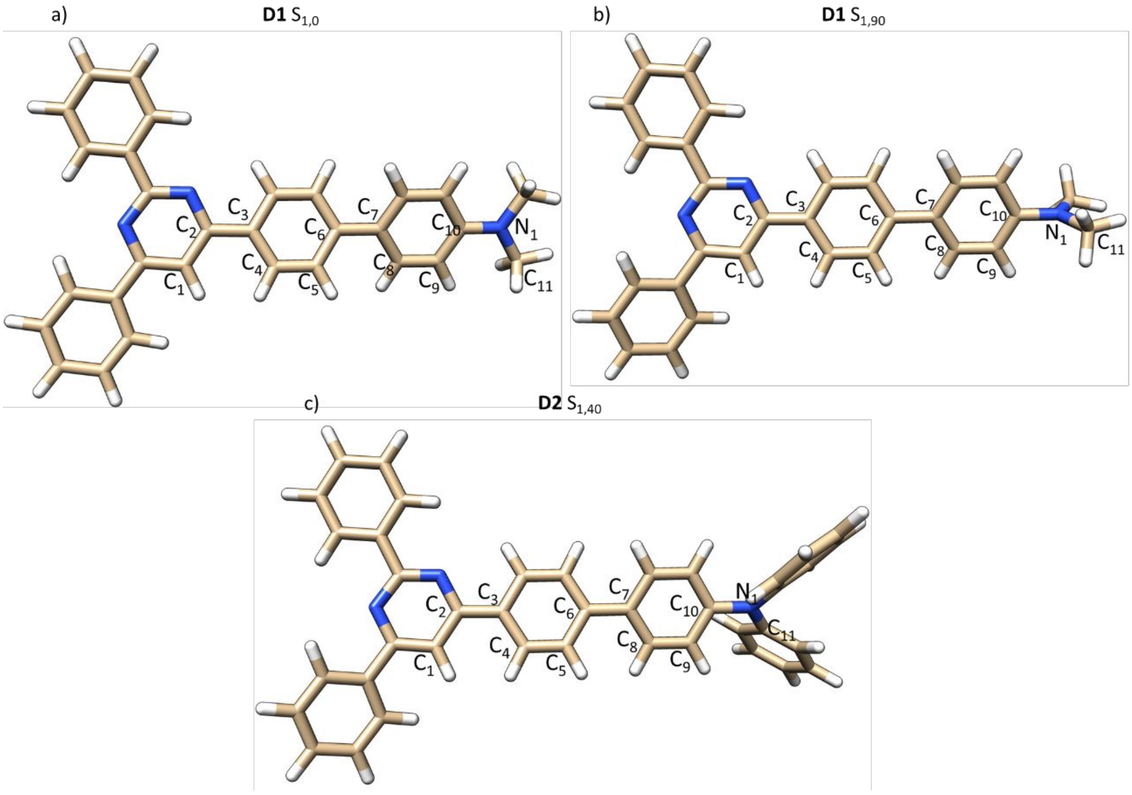

Solvent	Dihedral	D1		D2
		$S_{1,0}$	$S_{1,90}$	$S_{1,40}$
**HX**	$C_1C_2C_3C_4$	19.34	-2.06	-1.32
	$C_5C_6C_7C_8$	28.22	17.80	21.66
	$C_9C_{10}N_1C_{11}$	-1.51	89.93	54.83
**TOL**	$C_1C_2C_3C_4$	17.09	-1.93	-0.72
	$C_5C_6C_7C_8$	28.19	17.55	21.65
	$C_9C_{10}N_1C_{11}$	-3.15	89.94	51.71
**ACN**	$C_1C_2C_3C_4$	-3.35	-1.05	-2.30
	$C_5C_6C_7C_8$	24.22	16.11	16.62
	$C_9C_{10}N_1C_{11}$	-0.16	90.84	40.20
**DMSO**	$C_1C_2C_3C_4$	-3.22	-1.01	-2.30
	$C_5C_6C_7C_8$	24.09	16.09	16.50
	$C_9C_{10}N_1C_{11}$	-0.17	90.83	40.07

Figure S27. TD-DFT/B3LYP/6-311G**-optimized geometries calculated for the excited state (S_1) of **D1** (a and b) and **D2** (c). The values of the dihedral angles defining the optimized structures are given in the table expressed in degrees. Reproduced from

reference [1]. Copyright © 2023 Cortés-Villena, Soriano-Díaz, Domínguez, Vidal, Rojas, Aliaga, Giussani, Doménech-Carbó, Ortí, Galian and Pérez-Prieto. This is an open-access article distributed under the terms of the Creative Commons Attribution License (CC BY).

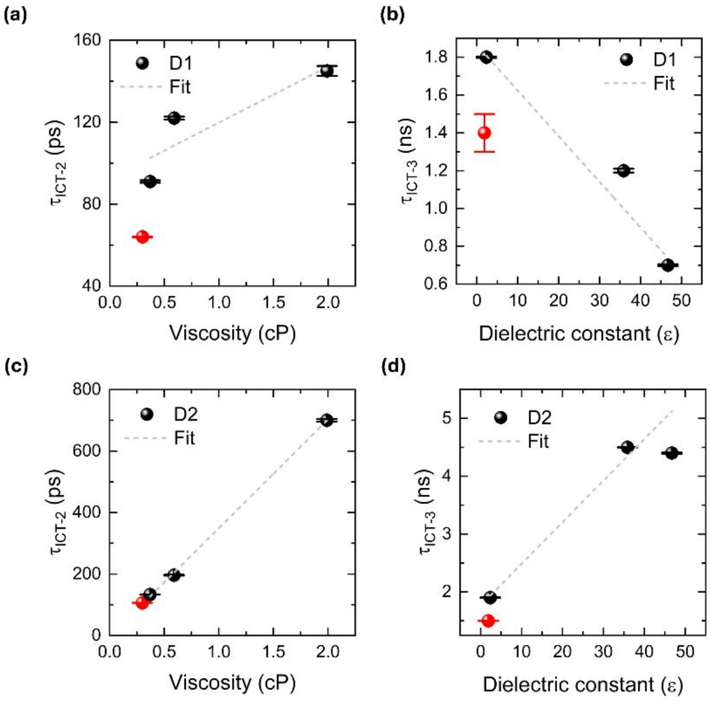

Figure S28. Relationship of τ_{ICT-2} and τ_{ICT-3} with (a) viscosity and (b) dielectric constant for **D1**. Relationship of τ_{ICT-2} and τ_{ICT-3} with (c) viscosity and (d) dielectric constant for **D2**. Red points indicate LE character of the S_1 state and therefore deviate from the expected trend.

References

1. Cortés-Villena, A.; Soriano-Díaz, I.; Domínguez, M.; Vidal, M.; Rojas, P.; Aliaga, C.; Giussani, A.; Doménech-Carbó, A.; Ortí, E.; Galian, R. E., et al., Governing the Emissive Properties of 4-Aminobiphenyl-2-Pyrimidine Push–Pull Systems Via the Restricted Torsion of *N*,*N*-Disubstituted Amino Groups. *Frontiers in Chemistry* **2023**, *11*, 1292541.
